# Slippery dopamine–fluoropolymer hybrid surface for improving biliary stent longevity

**DOI:** 10.1016/j.bioactmat.2026.02.003

**Published:** 2026-02-13

**Authors:** Tae Young Kim, Won-Jong Lee, Yurim Lee, Seo Jung Kim, Sungjin Min, Seyong Chung, Soo A Kim, Keun-Young Yook, Chang-Hwan Moon, Yeontaek Lee, Kijun Park, Dae-Hyun Kim, Jungmok Seo

**Affiliations:** a School of Electrical and Electronic Engineering, Yonsei University, 50-1 Yonsei-ro, Seodaemun-gu, Seoul, 03722, Republic of Korea; b Department of Veterinary Surgery, College of Veterinary Medicine, Chungnam National University, 99, Daehak-ro, Yuseong-gu, Daejeon, 34134, Republic of Korea; c Department of Pediatrics, Severance Children's Hospital, Yonsei University College of Medicine, Seoul, 03722, Republic of Korea; d Department of MetaBioHealth, Sungkyunkwan University, Suwon, 16419, Republic of Korea; e Department of Biopharmaceutical Convergence, Sungkyunkwan University, Suwon, 16419, Republic of Korea; f Division of Cardiology, Department of Internal Medicine, Severance Cardiovascular Hospital, Yonsei University College of Medicine, Seoul, 03722, Republic of Korea; g Department of Veterinary Surgery, College of Veterinary Medicine, Gyeongsang National University, 501, Jinjudae-ro, Jinju-si Gyeongsangnam-do, 52828, Republic of Korea; h Division of Engineering in Medicine, Department of Medicine, Brigham and Women's Hospital, Harvard Medical School, MA, 02139, USA

**Keywords:** Bile duct obstruction, Stent longevity enhancement, Stent blockage, Anti-biofouling coating

## Abstract

Biliary obstruction leads to bile retention and triggers a cascade of pathological events. Bile accumulation induces cholestasis and inflammation, progressing to hepatocellular injury, fibrosis, and ultimately liver failure. To restore bile flow, biliary stents are a necessary option due to their immediate patency. However, their high susceptibility to foreign body reaction (FBR) associated fibrosis, biofilm formation, and biliary sludge accumulation leads to frequent occlusion. To address this limitation, we developed the Enhanced Longevity by anti-fouling Functional coating for Stent (ELFS), a lubricant-infused coating that prevents stent occlusion. ELFS can be readily fabricated via a simple dip-coating solution process and employ a polydopamine (PDA) adhesion layer. Intravital imaging in mice confirmed that ELFS suppressed the FBR by blocking early neutrophil adhesion, which in turn prevented downstream immune-fibrotic cascades. At 3 h, neutrophil recruitment in the non-coated group was >20-fold higher than in ELFS-coated groups. Additionally, ELFS-coated stents remained free of biofilm for over six months in mice and maintained full open for two months in a rabbit common bile duct model. In contrast, non-coated stents resulted in complete occlusion, bile duct dilation (over 4 times), hepatomegaly (over 2 times), and fibrosis.

## Highlights

• Clinical challenge: Biliary stents often fail due to bile viscosity, foreign body reaction, fibrosis, and biofilm-driven occlusion.
• ELFS, a lubricant-infused antifouling coating, shows high mechanical stability without delamination under physiological flow.
• ELFS is fabricated via a simple dip-coating process using a PDA adhesion layer.
• ELFS reduced neutrophil recruitment by >20-fold, prevented biofilm for 6 months in mice, and maintained patency in rabbits.

## Graphical abstract

**Figure:**
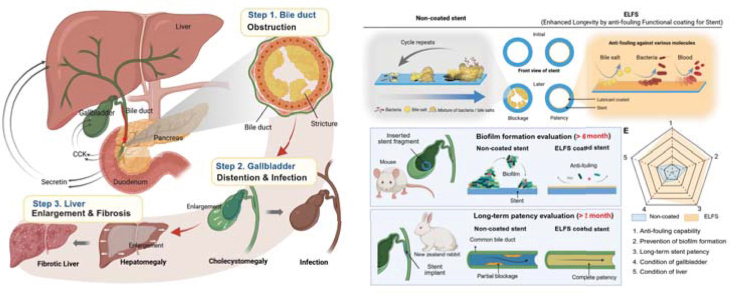

## 1 Introduction

Bile plays a crucial role in human physiology by facilitating the digestion and absorption of fats. It is also essential for the excretion of waste products, such as excess cholesterol and bilirubin, which helps regulate cholesterol levels and support detoxification [[1], [2], [3]]. These functions collectively contribute to metabolic homeostasis. However, various pathological conditions can disrupt bile flow, leading to obstruction of the bile duct [4]. These conditions can arise from multiple causes, including gallstone formation, inflammatory response, and malignancies (bile duct, pancreatic, or gallbladder cancer) [[5], [6], [7]], and they pose significant health risks due to their potential to cause serious complications. For example, obstruction-induced acute cholangitis has been associated with a mortality rate ranging from 10% to 30% despite advances in medical treatment [[8], [9], [10], [11]]. These statistics indicate the need for early intervention to prevent severe complications and improve patient outcomes.

To restore bile flow, several treatment options exist, including balloon dilation [12], percutaneous biliary drainage [13], biliary bypass surgery [14], and endoscopic placement of biliary stent. Among them, stent implantation is a prevalent and widely used approach due to its immediate relief of obstruction and ability to maintain bile flow over extended periods [15]. Two primary types of biliary stents are used in clinical practice: plastic and metal stents. Each type has distinct advantages and limitations, influencing their selection based on the patient's condition and treatment [16,17]. Metal stents, specifically self-expandable metal stents (SEMS), are widely preferred when long-term biliary drainage is required [18]. Their larger diameter and self-expanding properties help maintain patency for extended periods, significantly reducing the frequency of stent replacement [19]. Studies have shown that metal stents can maintain patience for up to 9 months, making them a more durable option compared to plastic stents [18,20,21]. However, despite these advantages, metal stents pose a major challenge: once deployed, they are difficult to remove. In addition, SEMS deployment can be technically challenging in cases with severely angulated or narrow biliary ducts [22,23]. In such anatomical complex cases, accurate positioning and expansion are difficult to achieve, which limits their applicability. In contrast, plastic stents are widely used due to their ease of placement, lower cost, and replaceability, making them ideal for short-term biliary drainage or cases where future intervention is planned [24]. However, a major drawback of plastic stents is their high susceptibility to occlusion and infection [25,26]. Their small diameter (1.65–4 mm) and non-expandable structure, combined with the plastic material's tendency to promote biofilm formation and biliary sludge accumulation, lead to frequent blockages [27]. According to previous reports, plastic stents typically require replacement every 2–4 months [28], with patency ranging from 77 to 126 days [29]. This frequent need for replacement not only increases the burden on patients but also raises the risk of complications and overall healthcare costs.

To overcome this, drug-eluting stents were developed to inhibit fibroblast proliferation and biofilm formation. However, their clinical efficacy was constrained by short drug-release duration and concerns regarding systemic toxicity. Another approach was silver nanoparticle (AgNP) coating to provide antibacterial and anti-inflammatory effects after radiofrequency (RF) ablation, aiming to reduce bacterial biofilm and tissue hyperplasia. However, these stents still suffered from issues such as burst Ag release, technical complexity, and limited long-term stability. To address these limitations, we introduced an anti-fouling coating strategy aimed at preventing biofilm formation and sludge deposition on plastic stents. Anti-fouling coatings are designed to minimize the adhesion of biological substances [30,31]. Among them, lubricant-infused anti-fouling coatings have emerged as a promising solution [32]. A key advantage of the coatings is their ability to self-repair through a liquid-wicking mechanism, ensuring defect-free performance. Despite these advantages, the application of lubricant-infused coatings remains limited due to the complexity of the coating process which requires substrates with nano- or microstructures [33]. Recent studies have reported lubricant-infused coatings that partially alleviate the need for nano- or microstructures by employing adhesive interlayers. However, these strategies have been primarily explored on simple planar substrates, and their extension to complex tubular geometries or functional medical devices has remained limited.

Herein, we present Enhanced Longevity by anti-fouling Functional coating for Stent (ELFS), featuring a lubricated surface designed to resist bio-substrates adhesion and prevent stent occlusion. By integrating a polydopamine (PDA) layer as an adhesion intermediate [34,35], ELFS overcomes the limitations of conventional complex coating methods. This approach enables stable and uniform application to long and tubular structures without requiring nano- or microstructures. Unlike previous studies that primarily demonstrated coating feasibility or proof-of-concept, ELFS exhibits robust durability under clinically relevant sterilization conditions. Importantly, ELFS exhibits exceptional robustness under clinically relevant sterilization and handling conditions, maintaining its structural integrity and functional performance after repeated autoclave sterilization (>9 cycles), ethylene oxide (EO) gas exposure (3 cycles), and gamma irradiation (3 cycles). This high level of sterilization tolerance underscores the suitability of ELFS for practical medical device applications, where repeated sterilization and strict safety requirements are unavoidable. Owing to their slippery surface, the stent presented excellent liquid-repellent properties against body and viscoelastic fluids. The anti-fouling properties against bacteria, blood, and bile were tested through a series of *in vitro* and *in vivo* conditions. To confirm its functionality, ELFS coating was applied to the inner surface of the plastic stent and subsequently implanted into both mouse and rabbit models. The mouse experiments revealed no biofilm formation on ELFS-coated stents during both short-term (one week) and long-term implantation (>6 months). In contrast, non-coated plastic stents showed biofilm formation in both periods, accompanied by mild elevations (about 1.5-fold) in liver enzymes indicative of hepatocellular stress. Furthermore, intravital imaging revealed that immune activation was initiated by a rapid surge of neutrophils, with more than a 20-fold increase observed in non-coated group compared to ELFS-coated group at 3 h. This early neutrophil response drove the subsequent cascade of foreign body reaction (FBR), ultimately leading to collagen encapsulation around the implant. Lastly, long-term patency of the stent was confirmed through implantation into the rabbit common bile duct. ELFS-coated stent fully open after 2 months with no signs of adverse effects, such as elevated bilirubin levels or hepatomegaly. In contrast, non-coated stent groups exhibited complete stent occlusion, which led to significant bile duct dilation (over 4 times enlargement), hepatomegaly (over 2 times enlargement), and liver fibrosis. Histological analysis revealed extensive collagen accumulation and liver function failure in non-coated stent group. These findings demonstrate that ELFS coating significantly enhances the longevity of implantable stents. We anticipate that this coating can be applied across a wide range of implantable medical devices, providing a promising solution to combat biofilm formation and occlusion.

## 2 Results and discussions

### 2.1 ELFS for bile duct stents

**Fig. 1:**
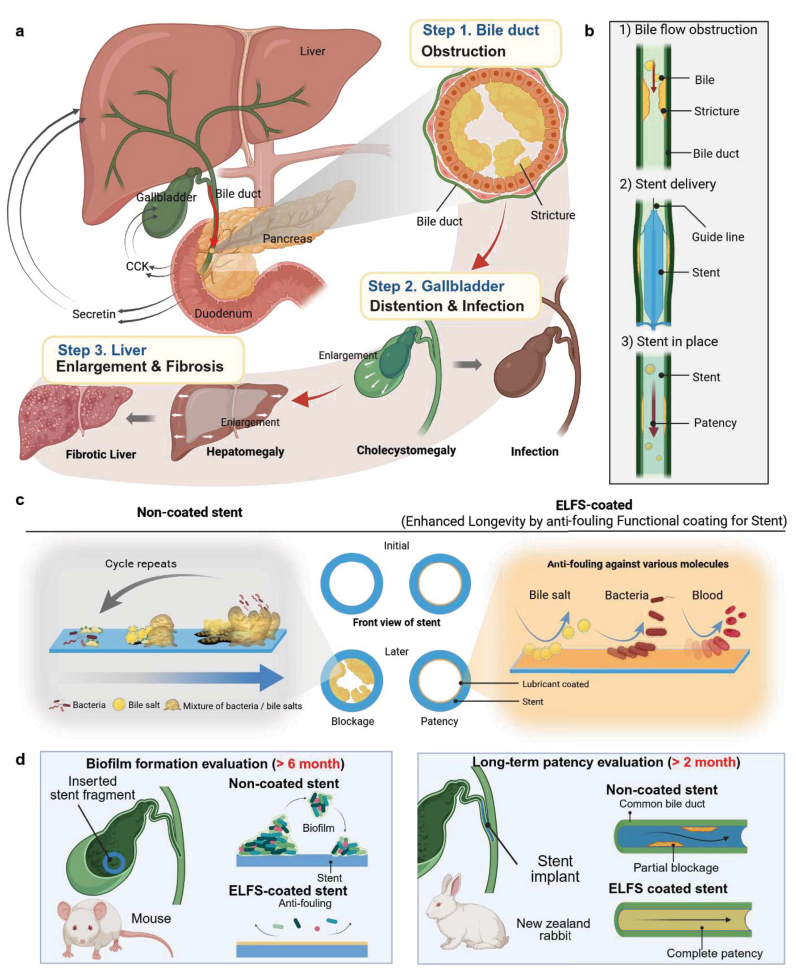
ELFS for bile duct stent. (a) Schematic illustration of complications affecting the bile duct, gallbladder, and liver due to the bile structure. (b) Schematic representation of the bile duct stent placement procedure. (c) Predicted outcomes of non-coated and ELFS-coated stents, showing the initial and later conditions. (d) Animal experiment to evaluate ELFS coating functionality: long-term (>6 months) biofilm formation tests in a mouse model and long-term (>2 months) patency evaluation with a rabbit model.

Fig. 1a illustrates a variety of complications induced by bile obstruction such as distension and infection of the gallbladder, enlargement of liver, and fibrotic liver formation. The bile flow is disrupted due to bile obstruction, which leads to cholestasis, inflammation, and metabolic disturbances [36,37]. Prolonged bile retention results in bile acids, bilirubin, and cholesterol accumulation within hepatocytes, which triggers cellular injury and initiates an inflammatory response [38]. As hepatocytes undergo necrosis, they release damage-associated molecular patterns (DAMPs). These signals recruit neutrophils to the injury site, leading to acute inflammation [39]. This inflammatory cascade causes tissue damage and contributes to the progression of cholestatic hepatitis. If bile obstruction persists, chronic inflammation develops, characterized by the sustained recruitment of lymphocytes and macrophages and the release of proinflammatory cytokines such as TNF-α and IL-1 [40,41]. This prolonged inflammatory state leads to periportal fibrosis. In this process, activated fibroblasts deposit excessive extracellular matrix (ECM) proteins around the portal areas [42,43]. As fibrosis progresses, hepatocytes attempt to compensate through a regenerative response characterized by proliferation and hypertrophy. However, the ongoing deposition of ECM proteins contributes to the structural remodeling of the liver, leading to progressive hepatomegaly [[44], [45], [46]]. Over time, chronic bile retention and persistent inflammation culminate in secondary biliary cirrhosis [47], where extensive fibrosis disrupts the normal liver architecture, leading to impaired hepatic function [48]. If left untreated, this condition may further deteriorate into hepatic failure, posing life-threatening consequences. The progression of cholestatic liver disease due to common bile duct obstruction follows a sequential pattern, involving bile stasis, acute and chronic inflammation, hepatomegaly, fibrosis, cirrhosis, and ultimately liver failure. To mitigate these complications, early intervention in bile duct obstruction is critical to preventing fibrotic remodeling and cirrhosis. To address bile strictures, stent placement procedures are commonly performed. This minimally invasive technique involves inserting a stent into the narrowed bile duct to restore normal bile flow and relieve obstruction (Fig. 1b). In many cases, this procedure provides immediate symptom relief. However, stents often become occluded due to various factors, including the high viscosity of bile, biofilm formation, and the accumulation of debris. To solve this issue, we have developed an anti-fouling coating using a lubricant, which is applied to the inner surface of the stent (Fig. 1c). ELFS is designed to prevent the adhesion of bile, bacteria, and blood. By applying ELFS, we aim to enhance the long-term patency of the stent, ensuring prolonged functionality and minimizing the need for frequent replacements or interventions. This innovative approach has the potential to significantly improve the efficacy of bile duct stenting and enhance patient outcomes. To evaluate the effectiveness of ELFS, we conducted *in vivo* experiments using both mouse and rabbits (Fig. 1d). In the mouse model, stent fragments were implanted into the gallbladder, and biofilm formation on the surface was monitored over 6 months. In the rabbit model, the stent was directly placed in the bile duct through a surgical procedure, and its long-term patency was observed over 2 months. These experiments provided crucial insights into the ability of ELFS to prevent biofilm accumulation and maintain bile duct patency. This demonstrates its potential as a promising solution for enhancing the durability and functionality of biliary stents.

### 2.2 Fabrication process of ELFS and characterization

**Fig. 2:**
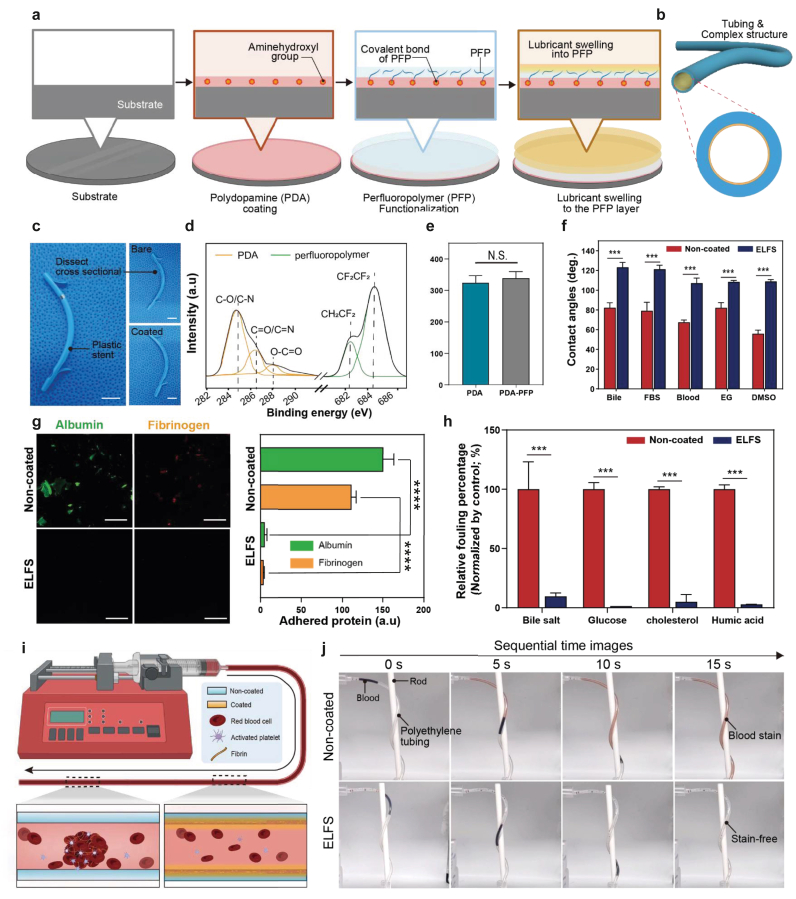
Fabrication process of ELFS and characteristics. (a) Schematic representation of the step-by-step fabrication process of ELFS. (b, c) Demonstration of the ELFS application on tubing and complex stent structures with corresponding optical images (scale bars, 1 cm). (d) Confirmation of chemical change induced by ELFS coating using XPS analysis. (e) Measurement of coating thickness of PDA alone and PDA-PFP with Dektak XT (n = 7). (f) CAs measurement of various liquids on non-coated and ELFS-coated polyethylene plate (n = 4). (g) Representative fluorescence images and quantitative analysis of plasma protein adhesion against non-coated and ELFS-coated substrates. Fluorescence intensities were background-subtracted, normalized to surface area, and expressed relative to the non-coated control (n = 4) (scale bars, 100 μm). (h) Relative fouling percentage of biomolecules (n = 5). (i, j) Schematic illustration and optical sequential photographs of blood staining experiment on non-coated, ELFS-coated polyethylene medical tubing. *(∗P < 0.05, ∗∗P < 0.01, ∗∗∗P < 0.001, and ∗∗∗∗P < 0.0001). ns, not significant.*

Fig. 2a illustrates the stepwise fabrication process of ELFS, from substrate preparation to final lubricant swelling. The process begins with the application of a PDA coating, which introduces amine-hydroxyl functional groups onto the surface. To achieve stable and uniform PDA coating, a previously reported oxidant-assisted deposition technique was employed to promote rapid and homogeneous formation of the PDA layer [49]. These groups facilitate the formation of amide bonds with the carboxyl groups of the perfluoropolymer (PFP), ensuring strong adhesion of the polymer layer (Fig. S1a). Once the PFP layer is securely anchored, a fluorinated lubricant is infused through swelling. This process forms a stable, slippery, and anti-fouling surface. A key advantage of this fabrication method is its ability to apply not only flat surfaces but also tubing and complex structures (Fig. 2b and c). The deposition of ELFS coating was first confirmed using X-ray photoelectron spectroscopy (XPS), which verified the successful deposition of PDA onto the surface, as well as the subsequent conjugation of PFP (Fig. 2d and Fig. S1b). The thickness of the ELFS coating was quantified after formation of the PDA and PDA-PFP framework. As shown in Fig. 2e, the coating exhibited a uniform thickness of approximately 300–350 nm across the substrate. Importantly the subsequent lubricant infusion step does not generate an additional discrete layer but instead induces swelling within the PFP network. Accordingly, the measured thickness predominantly represents the underlying structural coating framework. Consistent with the profilometry results, scanning electron microscopy (SEM) images further confirmed the uniform coverage of the ELFS coating over the substrate (Fig. S1c). In addition, SEM cross-sectional analysis revealed a coating thickness comparable to that measured by Dektak profilometry, further validating the uniformity and thickness of the ELFS coating (Fig. S1d). These results confirm the formation of a conformal and uniform ELFS coating suitable for application to plastic stent surfaces. Following the swelling of the fluorinated lubricant, we assessed the coating's repellency by measuring the contact angles (CAs) of various solvents, including bile, fetal bovine serum (FBS), blood, ethylene glycol (EG), and dimethyl sulfoxide (DMSO). The results demonstrated that ELFS maintained high repellency regardless of the surface tension of the solvent (Fig. 2f). To confirm anti-biofouling properties, we examined albumin and fibrinogen, which readily adhere to medical implants. On non-coated surfaces, these proteins exhibited significant adhesion. In contrast, polyethylene glycol (PEG) -coated surfaces widely used as an anti-fouling coating showed only partial suppression. However, ELFS-coated surfaces remained entirely free of protein attachment, demonstrating superior anti-fouling performance. The fluorescence signals of the adhered proteins were quantified for statistical analysis (Fig. 2g and Fig. S2a and b). To validate the anti-fouling performance of ELFS under physiologically relevant conditions, we examined its resistance to bile, glucose, cholesterol, and humic acid. The results showed that ELFS effectively repelled all tested substances, demonstrating their exceptional anti-fouling properties across a wide range of biomolecules (Fig. 2h). We compared PEG-coated and ELFS-coated surfaces using blood. PEG-coated surfaces were entirely stained by blood, whereas ELFS-coated surfaces remained completely clean and stain-free (Fig. S2c). To assess performance in a long tubular geometry, we used PE tubing that matches the plastic stent material. We perfused horse blood at a constant flow to examine the coating resistance to blood fouling (Fig. 2i). To deconvolute the contribution of each coating component, additional control groups were evaluated, including PDA-only, PFP-only, and lubricant-free PFP coatings (Fig. S2d). In the PDA-only group, blood staining was readily observed, consistent with the known protein-adhesive nature of PDA surfaces. In the PFP-only group, blood fouling was also evident, likely due to insufficient coating stability in the absence of an adhesive interlayer. For the lubricant-free PFP group, the presence of the PDA interlayer enabled stable PFP deposition; however, only partial resistance to blood fouling was observed. This indicates that the PFP framework alone is insufficient to achieve a fully non-fouling interface. In contrast, ELFS-coated tubing remained almost completely stain-free throughout the perfusion period, whereas non-coated tubing became entirely covered with blood stains (Fig. 2j). These comparative results showed the synergistic role of the PDA adhesion layer, PFP framework, and infused lubricant in achieving robust anti-fouling performance in tubular geometries. Collectively, these findings suggest that ELFS provides a more effective strategy for maintaining lumen cleanliness and coating integrity under flow conditions relevant to biliary stents.

### 2.3 ELFS's stability under various conditions

**Fig. 3:**
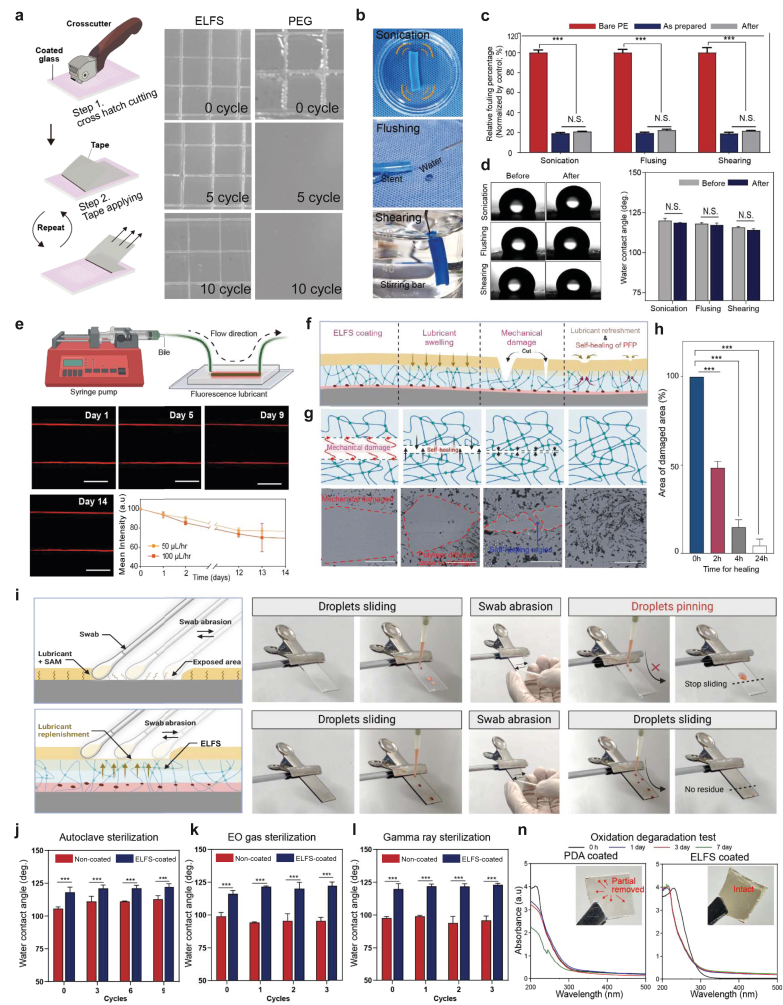
ELFS's stability under various conditions. (a) Schematics of cross-hatch cutting test (ISO 2409) and optical images of ELFS and PEG coating after hatch cutting test (n = 3). (b-d) Stability of ELFS coating against body fluidic proteins (humic acid), and CA measurements following different durability tests, including 7 days of perpendicular water flushing, 1 day of ultrasonication without temperature control, and 30 days of water shearing (n = 4). (e) Stability evaluation of ELFS coating under continuous bile flow conditions using red fluorescence-dyed ELFS-coated microfluidic devices, with quantitative analysis performed using ImageJ software (n = 4). (f-h) Time-sequential representative images showing damage and self-healing of ELFS through heat-induced viscoelastic reflow and its corresponding quantitative analysis using ImageJ (n = 4) (scale bars, 100 μm). (i) Schematic representation of the swab abrasion test and the lubricant replenishment mechanism of ELFS. Time-lapse images illustrate the sliding behavior of water droplets on inclined surfaces before and after swab abrasion (n = 4). (j-l) Evaluation of ELFS coating stability after common medical sterilization methods, including autoclave, ethylene oxide (EO) gas, and gamma-ray sterilization, assessed by static DI water CAs measurements (n = 3), confirming its suitability for implantable medical devices. (n) Confirmation of the coating degradation under oxidative conditions and corresponding UV-visible spectra measurement (n = 3). *(∗P < 0.05, ∗∗P < 0.01, ∗∗∗P < 0.001, and ∗∗∗∗P < 0.0001). ns, not significant.*

For practical applications, the mechanical robustness of the coating is crucial to ensure strong adhesion to the target substrate. To assess the mechanical stability of ELFS, we performed a standardized adhesion test following ISO 2409 guidelines, using a crosscut test to evaluate its durability under stress (Fig. 3a). ELFS coated substrates were compared against PEG coating, which is utilized in various applications due to its biocompatibility and anti-fouling capability. During the test, each sample underwent crosscutting using a specialized cutter with six evenly spaced blades (1 mm apart), creating a grid of 25 small squares within the coating. After making the cuttings, adhesive tape was applied and peeled off horizontally to assess coating retention. Optical images revealed a difference between the two coatings: the PEG coating initially maintained intact after crosscutting but delamination was observed upon repeated tape removal, indicating weak adhesion. In contrast, ELFS coating exhibited exceptional adhesion, with no observable detachment of the coatings. This strong mechanical stability is attributed to the robust amide (RCONR) bonding between PDA and PFP, which significantly reinforces the coating's adherence to the substrate. To further investigate the mechanical durability of ELFS under different aqueous external stresses, additional tests were conducted (Fig. 3b). ELFS-coated substrates were subjected to three challenging conditions including ultrasonication in a water bath, perpendicular water flushing, and water shear flow. Following these mechanical stress tests, we performed a quantitative analysis to evaluate fouling resistance. For this, we used humic acid, a biologically relevant molecule commonly found in the ECM of the human body. To test the anti-fouling performance, the substrates were incubated in a humic acid suspension (5 mg/mL in PBS solution) for 24 h. UV-vis absorbance measurements (200–254 nm) were then conducted to assess surface contamination. The results showed that ELFS-coated stents exhibited nearly identical fouling resistance before and after exposure to all three stress conditions (Fig. 3c). Furthermore, we measured static water CAs to evaluate anti-fouling capability before and after experiments. The negligible changes in CA indicates that the coating remained mechanically intact and that the lubricant infused structure was retained under aqueous stresses (Fig. 3d). Static wetting measurements alone do not capture the mechanical challenges imposed by bile flow *in vivo*, where interfacial shear can progressively destabilize lubricant-infused surfaces. Although bile flow *in vivo* is intermittent rather than constant, the present *in vitro* setup was not intended to fully replicate the physiological flow profile. Instead, constant bile flow was applied to ensure sufficient cumulative shear exposure over time, enabling robust evaluation of lubricant retention under sustained mechanical stress. Accordingly, the retention of the lubricant-infused layer was assessed using a microfluidic polydimethylsiloxane (PDMS) device at flow rates of 50 and 100 μL h^−1^. To further enhance physiological relevance, bile extracted directly from rabbits was used as the perfusion medium, preserving native bile components such as bile acids, proteins, and endogenous enzymes. Notably, after fourteen days of continuous bile flow, more than 80% of the lubricant remained intact (Fig. 3e). This indicates that ELFS maintains its structural integrity and functional performance over extended periods in fluidic conditions. Anti-biofouling implant materials are often exposed to mechanical stresses such as scratching, which can occur during device sterilization, or handling prior to implantation, and may compromise their surface functionality. ELFS has the ability to soften when heated above PFP's glass transition temperature (Tg). This softening is attributed to the increased free volume within the PFP polymer matrix, enhancing its mobility. As a result, ELFS can self-heal by allowing the viscous reflow of PFP to cover damaged areas (Fig. 3f). To demonstrate the self-healing capability of ELFS, time-dependent optical images were captured at 0, 2, 4, and 24 h after thermal treatment (Fig. 3g and h). The thermal condition was selected to activate polymer mobility within the coating, allowing the self-healing process to be visualized under controlled conditions. When a scratch occurs, the coating is disrupted, creating separate interfaces. Raising the temperature close to its Tg induces viscoelastic reflow and enhances chain mobility. This facilitates interfacial diffusion between the damaged regions and promotes structural reorganization, ultimately restoring coating continuity. Such thermally activated recovery behavior is relevant to elevated-temperature conditions encountered during device processing and sterilization workflows, indicating that ELFS can tolerate and recover from mechanical damage without loss of structural integrity. The optical images clearly illustrate the time-dependent self-healing behavior of the coating. Additionally, the liquid-repellent performance of ELFS under mechanical stress was evaluated using a cotton swab abrasion test (Fig. 3i). Substrates coated with ELFS and fluorinated self-assembled monolayers (F-SAM) were subjected to the abrasion test. Prior to abrasion, both samples were immersed in the lubricant for 4 h. For each sample, a minimum of 20 swab strokes were applied to remove the lubricant from the surface. Sequential images in Fig. 3i show red-color dyed water droplet behavior after abrasion. On the SAM-coated surface, lubricant removal resulted in water droplets being pinned to the abraded area, indicating a degradation of anti-biofouling properties. In contrast, the lubricant-swollen surface of ELFS remained mechanically robust, retaining its liquid-repellent properties even after extensive abrasion. Water droplets continued to slide smoothly without pinning, demonstrating the superior durability of ELFS coating. The mechanical robustness of ELFS is attributed to the amorphous nature of PFP, which enhances wear resistance and its lubricant-swelling properties. Steam-based autoclave sterilization is widely utilized in industrial and healthcare settings to eliminate harmful substances before clinical applications. Static CAs and anti-fouling properties with albumin were confirmed before and after each cycle to assess any potential degradation in fouling resistance (Fig. 3j and Fig. S2e). In addition to autoclaving, the coated samples were subjected to ethylene oxide (EO) gas sterilization and gamma-ray sterilization (Fig. 3k and l). After every sterilization cycle, CAs measurements were taken to determine any changes in surface properties. Remarkably, ELFS-coated surfaces demonstrate almost identical CAs before and after all sterilization processes. Several studies have reported that PDA coatings undergo degradation in physiological environments over time due to oxidative enzymes. To evaluate the stability of ELFS coating under such conditions, we conducted an *in vitro* ROS-mediated degradation assay. For this, only PDA-coated and ELFS-coated glass substrates were used to simulate an enzyme-derived oxidative environment (Fig. S3a). The UV-Vis absorbance data in Fig. 3n, the PDA-coated group exhibited a gradual decrease in absorbance over time, indicating progressive degradation of the coating. After 7 days, the absorbance was reduced by nearly half, confirming significant degradation of the PDA layer. In contrast, ELFS-coated group maintained stable absorbance levels throughout the 7 days. CA's measurements were consistent with UV-Vis absorbance data (Fig. S3b and c). This stability can be attributed to the protective effect of the PFP layer and infused lubricant, which effectively shielded the PDA layer from enzymatic degradation.

### 2.4 *In vitro* cell evaluations

**Fig. 4:**
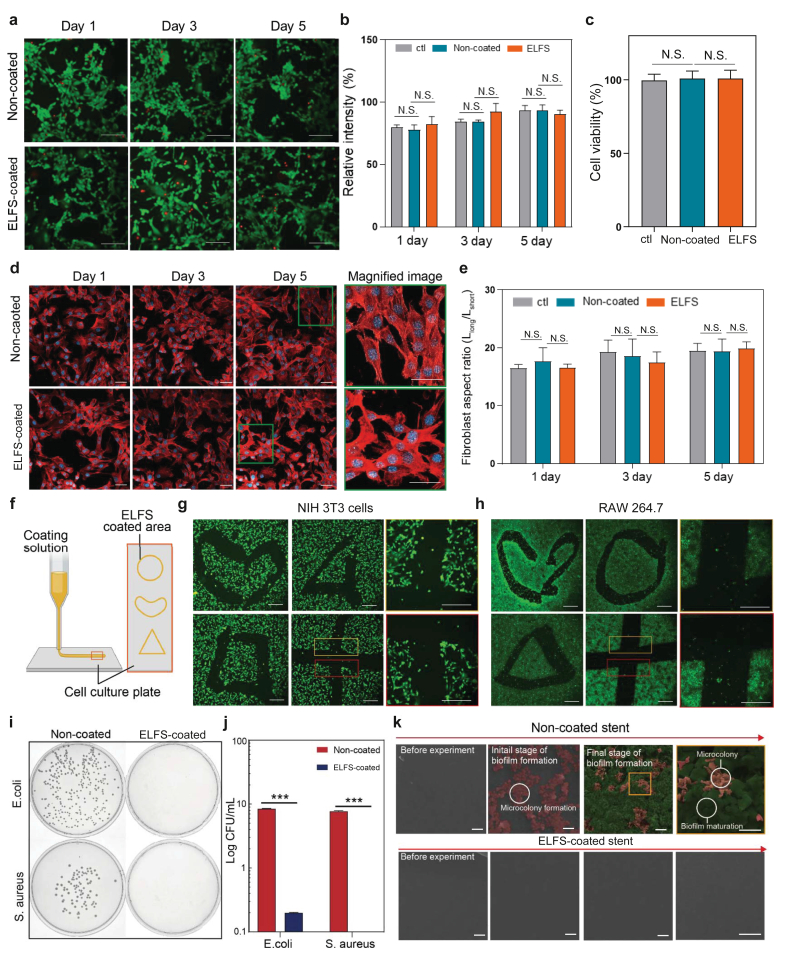
*In vitro* cell evaluations. (a, b) Fluorescence microscopic images of NIH 3T3 cells stained with a live/dead kit and corresponding quantitative analysis (n = 4) (scale bars, 100 μm). (c) Cytotoxicity analysis with NIT-3T3 cells using CCK-8 kit (n = 4). (d, e) Morphological analysis of NIH 3T3 cells stained for actin (red) and nucleus (blue), with fibroblast aspect ratio analysis (scale bars, 100 μm) (n = 4). (f) Schematic illustration demonstrating the selective application of ELFS coating to the target region. (g, h) Fluorescence images showing selective adhesion of NIH 3T3 and RAW 264.7 cells to ELFS-uncoated region (n = 4) (scale bars, 100 μm). (i, j) Optical images and quantification of adhered colony-forming units (CFUs) on non-coated and ELFS-coated plates after incubation in *E. coli* and *S. aureus* suspensions for 24 h (n = 4). (k) Sequential SEM images depicting biofilm formation on non-coated and ELFS- coated stent fragments (n = 3) (scale bars, 0.5 μm). *(∗P < 0.05, ∗∗P < 0.01, ∗∗∗P < 0.001, and ∗∗∗∗P < 0.0001). ns, not significant.*

To evaluate the biocompatibility of the ELFS coating, ELFS-coated stent fragments were incubated with NIH 3T3 fibroblasts and human biliary epithelial cells using a transwell system for five days. Cell viability was assessed through a live/dead assay and a CCK-8 assay. Fluorescence images obtained from the live/dead assay were quantified, revealing no statistically significant difference in fluorescence intensity among control, non-coated, and ELFS-coated groups. This indicates that ELFS coating does not induce significant cytotoxicity (Fig. 4a and b, and Fig. S4a and b). Similarly, the CCK-8 assay confirmed that ELFS-coated stents had no adverse effects on cells (Fig. 4c). In addition to cell viability, morphological changes in NIH 3T3 fibroblasts were analyzed to further assess biocompatibility. Changes in cell morphology can serve as a biomarker for evaluating material-induced cellular responses. Fibroblasts undergoing inflammatory activation tend to transition from their natural spindle shape to a rounded form due to cytoskeletal remodeling. To investigate this, actin filaments were stained using TRITC phalloidin, allowing for the visualization of structural alterations (Fig. 4d, and Fig. S4a). The aspect ratio (L_long_/L_short_) of the fibroblasts was quantified from fluorescence images taken after five days of exposure to stent fragments (Fig. 4e). The results showed no significant difference in cellular morphology among control, ELFS-coated, and non-coated stent fragment groups. One of the key characteristics of ELFS coating is its ability to be selectively applied to specific areas. To confirm this property, a syringe was used to apply ELFS in the shapes of a heart, triangle, square, and cross onto the surface of a cell culture dish (Fig. 4f). After coating, NIH 3T3 cells and RAW 264.7 cells were cultured on the dish for five days (Fig. 4g, h and Fig. S4c and d). Following the incubation period, the cells were stained using a live/dead kit to assess cell adhesion. The results showed that cells did not adhere to ELFS-coated regions, whereas they selectively adhered only to non-coated areas. Furthermore, we examined the anti-biofouling properties of ELFS using two representative infectious pathogenic bacteria: gram-negative *Escherichia coli (E. coli)* and gram-positive *Staphylococcus aureus (S. aureus).* These bacteria are among the most common culprits of biofilm formation and can ultimately lead to surgical site infections. Optical microscope images and colony-forming unit (CFU) measurements were used to analyze bacterial adhesion on different surfaces (Fig. 4i and j). ELFS coating demonstrated exceptional anti-fouling performance, with virtually no bacterial attachment observed after 24 h of incubation. Additionally, we experimented to determine whether biofilm formation occurred (Fig. 4k). Non-coated stent fragment exhibited the early stages of biofilm development, beginning with microcolony formation and progressively advancing to the final stage of biofilm maturation. In contrast, ELFS-coated group showed no bacterial adhesion or biofilm formation throughout the same period. These results further confirm the strong anti-biofouling properties of ELFS coating, effectively preventing bacterial accumulation and biofilm development.

### 2.5 In vivo evaluation of the stepwise FBR to ELFS coating

**Fig. 5:**
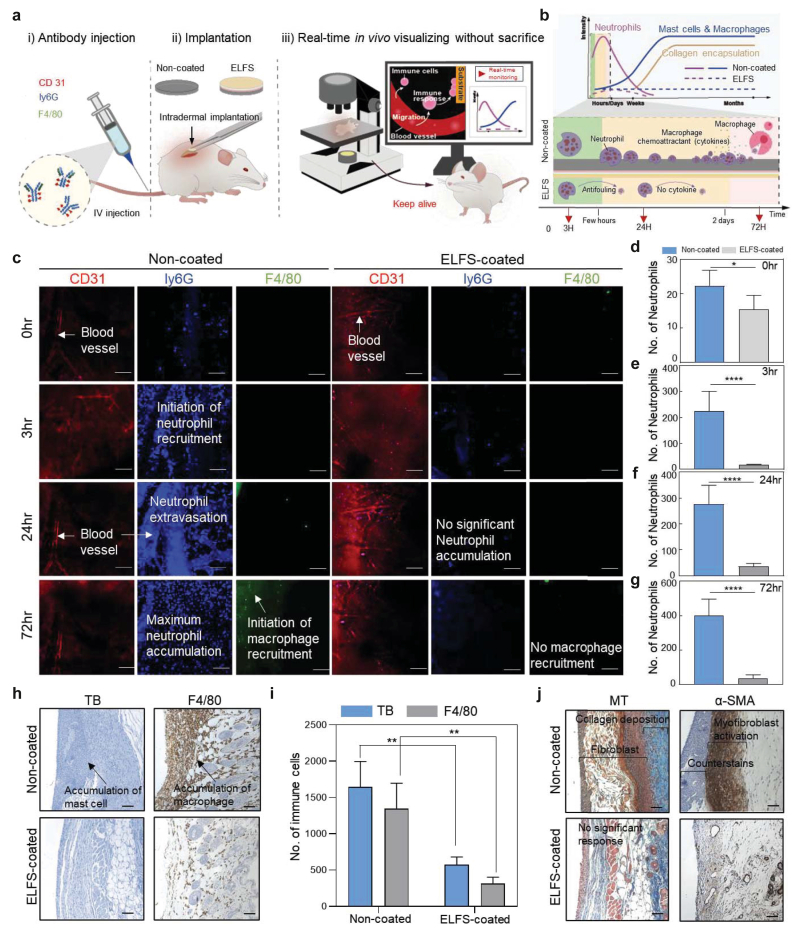
*In vivo* evaluation of the stepwise FBR to ELFS coating. (a) Schematics of the experimental workflow. (b) Conceptual timeline of the typical FBR (neutrophil infiltration and cytokine release, macrophage and mast cell responses, and collagen encapsulation) and its attenuation with ELFS coating. (c) Representative immunofluorescent images of vascular endothelial cells (CD31, red), neutrophils (Ly6G, blue), and macrophages (F4/80, green) at 0 h, 3 h, 24 h, and 72 h (n = 4) (scale bars, 100 μm). (d-g) Quantification of neutrophil counts at the indicated time points (n = 4). (h, i) Histological analysis after one week of implantation using TB and F4/80 staining image and its corresponding quantification (n = 4) (scale bars, 100 μm). (j) Evaluation of fibroblast collagen deposition, and myofibroblast activation using MT and α-SMA staining (n = 4) (scale bars, 100 μm). *(∗P < 0.05, ∗∗P < 0.01, ∗∗∗P < 0.001, and ∗∗∗∗P < 0.0001). ns, not significant.*

To confirm the sequence of host responses following stent implantation, we performed intravital imaging combined with histological analyses. Once implanted, the stent surface becomes rapidly covered by plasma proteins (e.g., fibrin and albumin), which subsequently promote cellular adhesion, ECM deposition, and ultimately collagen accumulation. To directly visualize these events *in vivo*, we used intravital imaging (Fig. 5a). This microscopy technique allows us to observe biological processes in real time within living animals under natural physiological conditions. To observe the overall FBR process, non-coated and ELFS-coated polyethylene (PE) films were implanted intradermally into the dorsal skin of mice being maintained alive throughout the imaging period. Although the biliary system is the intended clinical application of ELFS, direct intravital imaging of biliary implants is technically challenging due to anatomical inaccessibility and motion artifacts. Therefore, intradermal implantation in the dorsal skin was employed as a practical and well-established model to visualize early-stage FBR dynamics, which are largely conserved across tissues and are particularly relevant for assessing initial immune cell adhesion and activation. Fig. 5b schematically illustrates the typical temporal cascade of the FBR, beginning with neutrophil infiltration and cytokine release, progressing through macrophage recruitment, mast cell activation, and culminating in collagen encapsulation. In contrast, ELFS-coated implants are anticipated to elicit attenuated immune responses due to their anti-fouling characteristics. Intravital imaging revealed a marked difference between non-coated and ELFS-coated implants (Fig. 5c). In non-coated group, neutrophils labeled with Ly6G (blue) were progressively recruited to the implantation site, beginning as early as 3 h. We observed clear evidence of neutrophil extravasation at 24 h, as the regions marked by CD31 (red), indicative of blood vessels, were densely filled with Ly6G^+^ signals, consistent with neutrophil accumulation within the vasculature and subsequent extravasation toward the implant site. At 72 h, neutrophils reached maximal accumulation, indicating sustained inflammatory activation. At this time (72 h), macrophages labeled with F4/80 (green) also appeared, which demonstrates the transition from acute to chronic inflammatory responses. By contrast, ELFS-coated implants exhibited no significant neutrophil accumulation throughout the entire observation period. These findings suggest that the absence of early neutrophil activation in ELFS-coated samples prevents downstream macrophage infiltration. Quantitative analyses of neutrophil infiltration at each time point (Fig. 5d–g) confirmed these observations, showing a gradual and significant increase in neutrophil numbers in the non-coated group, whereas ELFS-coated implant maintained consistently low levels across all time points. To further evaluate the host response, we analyzed the tissue one week after implantation (Fig. 5h–j). In non-coated group, toluidine blue (TB) staining revealed a marked increase in mast cells around the implant surface (Fig. 5h). Mast cells are normally resident in skin tissue, but their population markedly expands in response to inflammatory stimulation. We observed a marked increase in mast cells in non-coated group, as demonstrated by TB staining. This accumulation was accompanied by a subsequent increase in macrophages, as confirmed by F4/80 immunostaining, which demonstrated dense macrophage infiltration in the implant region. Quantitative analysis confirmed that the number of mast cells and macrophages was significantly higher in non-coated group compared to ELFS-coated group (Fig. 5i). To further evaluate fibrotic tissue responses, we performed Masson's trichrome (MT) staining. This histological analysis revealed a thick layer of fibroblast-rich tissue and extensive collagen deposition forming a fibrotic capsule around non-coated implants, whereas ELFS-coated implants exhibited no significant fibrotic encapsulation (Fig. 5j). Consistent with the MT staining, α-Smooth Muscle Actin (α-SMA) immunohistochemistry showed abundant α-SMA positive myofibroblasts surrounding non-coated implants, whereas ELFS-coated implants exhibited negligible staining (Fig. 5j). These results are consistent with a stepwise FBR, in which early neutrophil recruitment is associated with subsequent immune cell infiltration and fibrotic tissue formation. Importantly, the ELFS-coated implants showed reduced neutrophil accumulation along with lower macrophage recruitment, myofibroblast activation, and fibrotic encapsulation, consistent with an attenuated FBR cascade. This suppression was further corroborated by albumin adhesion tests. The fluorescence imaging showed substantial albumin adsorption on non-coated stents, whereas ELFS-coated stents exhibited minimal albumin attachment (Fig. S4e and f). In addition, CA measurements before and after 1 week of implantation demonstrated that ELFS coating largely retained its anti-fouling property *in vivo* (Fig. S5g). To assess the *in vivo* biocompatibility and local tissue stability of the ELFS coating, histological analyses were performed following subcutaneous implantation. ELFS-coated PE films were implanted subcutaneously in mice, which were sacrificed 7 days post-surgery, with sham-operated mice used as controls (Fig. S4h). Hematoxylin and eosin (H&E) staining of tissues adjacent to the implanted films revealed no signs of inflammation, necrosis, or abnormal tissue responses, indicating good *in vivo* biocompatibility of the ELFS coating.

### 2.6 Long-term biofilm formation in a mouse model

**Fig. 6:**
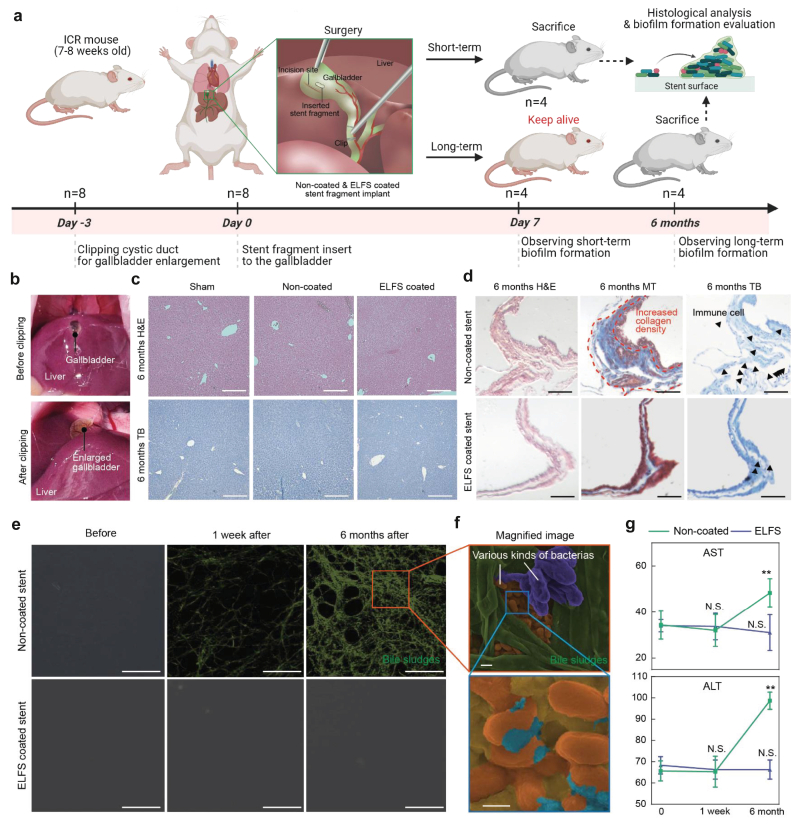
Long-term biofilm formation in a mouse model. (a) Schematic illustration of long-term biofilm formation test using a mouse model. (b) Representative optical image of the liver before and after bile duct clipping. (c, d) Histological analysis of liver and gallbladder after six months of implantation using H&E and TB staining (n = 3) (scale bars, 100 μm). (e) Sequential SEM images depicting bile deposition on non-coated and ELFS-coated stent fragments (n = 3) (scale bars, 1 μm). (f) Magnified image of an orange square box of Fig. 6e. (scale bars, 0.5 μm). (g) AST and ALT level measurement throughout the experimental period (n = 3). *(∗P < 0.05, ∗∗P < 0.01, ∗∗∗P < 0.001, and ∗∗∗∗P < 0.0001). ns, not significant.*

Biliary stents are continuously exposed to bile-rich environments, where prolonged exposure can lead to surface conditioning and bile stasis that facilitate bacterial adhesion and biofilm formation; therefore, effective suppression of biofilm formation is critical for maintaining long-term stent function. We investigated whether biofilm formation occurs on the surface of stents when exposed to bile for a long period using a mouse model. Direct implantation into the mouse bile duct is technically infeasible due to its extremely small diameter; therefore, stent fragments were implanted into the gallbladder, a bile-rich and relatively static environment suitable for biofilm evaluation. A small incision was made in the gallbladder of the mice, followed by the insertion of either non-coated or ELFS-coated stent fragment (Fig. 6a). The implanted stent fragments were examined one week and six months post-implantation to observe the stents' surface changes. To facilitate surgery, the common bile duct was clipped to enlarge the gallbladder, which increased its size by approximately twofold compared with the native state (Fig. 6b). The stents were inserted into the enlarged gallbladder of the mice and sacrificed after one week and 6 months. Histological examinations of the liver using H&E and TB staining revealed no significant differences between non-coated and ELFS-coated groups (Fig. 6c). By contrast, in the gallbladder, non-coated group exhibited a marked increase in collagen density, indicating progressive structural degradation over time (Fig. 6d). This discrepancy arises from the distinct microenvironments of the liver and gallbladder. The liver is highly vascularized and possesses strong regenerative capacity, which may buffer local tissue damage and explain the absence of major structural alterations. In contrast, the gallbladder represents a confined space continuously exposed to bile components and mechanical irritation from the implanted stent. SEM imaging was performed to examine the surface morphology of the implanted stents. In non-coated group, bile sludge accumulation was evident soon after one-week post-implantation, with a substantial increase observed after six months (Fig. 6e and Fig. S4i). High-magnification SEM analysis further revealed the presence of bacterial colonies, confirming the biofilm-like accumulation on non-coated stent surfaces (Fig. 6f). In contrast, ELFS-coated stents exhibited a marked reduction in bile sludge accumulation, attributed to the anti-biofouling properties of ELFS coating. ELFS coating effectively inhibited both bile sludge deposition and biofilm-like accumulation, demonstrating its potential to enhance long-term stent patency in bile-exposed environments. Additionally, aspartate aminotransferase (AST) and alanine aminotransferase (ALT) levels were measured to evaluate potential liver toxicity (Fig. 6g). A moderate elevation in liver enzyme levels was observed in non-coated stent group following long-term implantation. Severe hepatotoxicity is generally defined as 5-fold elevations in ALT. However, the rise in non-coated group was limited to about a 1.5-fold increase in ALT, consistent with mild hepatocellular stress without histological alterations. This discrepancy may be attributed to the higher sensitivity of serum transaminases, which can reflect subtle or early hepatobiliary stress even in the absence of apparent histological alterations. In the non-coated stent group, persistent biofilm formation and bile sludge accumulation may have contributed to chronic biliary burden. This secondary stress on hepatocytes could result in mild enzyme leakage without inducing overt tissue remodeling. Collectively, these observations suggest that anti-biofouling coatings may help reduce biofilm-associated biliary stress during long-term stent implantation, rather than indicating direct liver injury.

### 2.7 Evaluation of long-term patency with the rabbit model

**Fig. 7:**
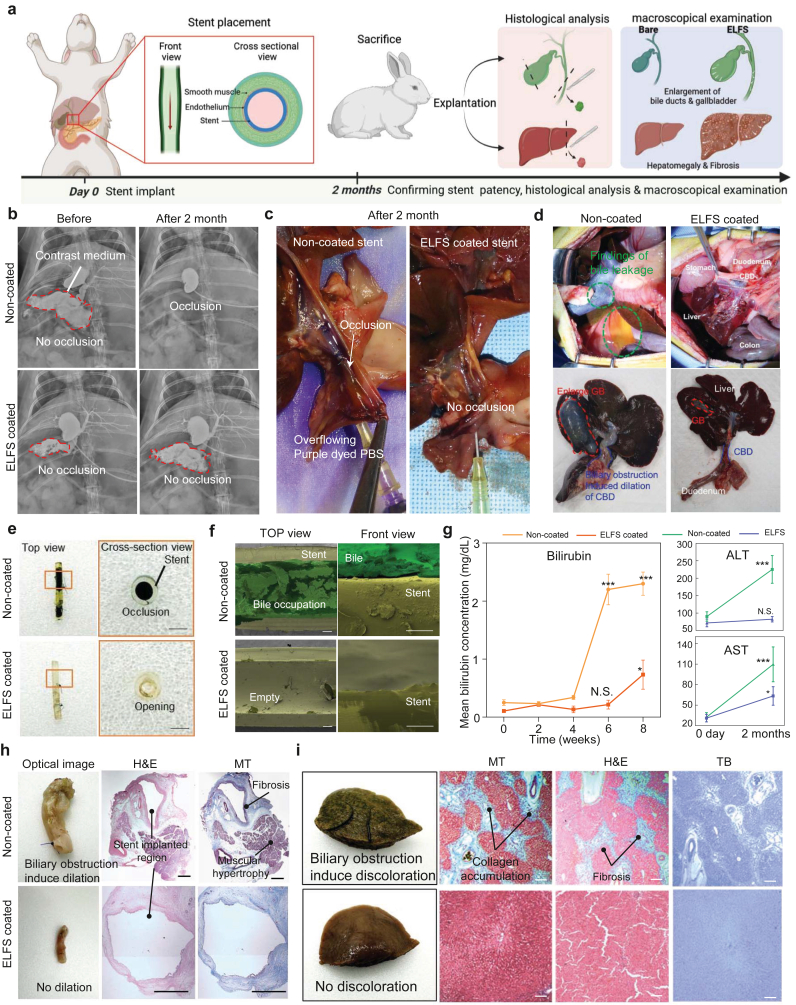
Evaluation of long-term patency with rabbit model. (a) Schematic representation of the stent placement procedure, experimental plan, and analysis methods. (b) Representative X-ray photographs demonstrating stent patency over time. (c) Confirmation of stent patency using explanted liver and bile duct perfused with purple-dyed PBS. (d) Macroscopic examination assessing complications caused by bile accumulation and overflow due to bile stricture. (e) Representative optical images of the stents after two months, with cross-sectional views showing bile-filled stents in non-coated group, while ELFS-coated stents remained fully open (scale bars, 0.5 mm). (f) Corresponding SEM image of the stents shown in Fig. 6e (scale bars, 100 μm and 10 μm, respectively). (g) Measurement of bilirubin, ALT, and AST levels in the blood throughout the experimental period (n = 4). (h, i) Representative optical and histological images of the bile duct and liver. (scale bars, 100 μm). Optical photographs of bile after 2 months of experiments and measurements of its viscosity. (n = 4). *(∗P < 0.05, ∗∗P < 0.01, ∗∗∗P < 0.001, and ∗∗∗∗P < 0.0001). ns, not significant.*

We evaluated the effectiveness of ELFS coating by applying it to a stent lumen and implanting it into the common bile duct of a rabbit to observe long-term patency. Rabbits were selected as an *in vivo* model because biliary stent implantation can be performed without pre-inducing liver disease. In addition, the relatively small bile duct diameter provides a stringent condition that enables sensitive evaluation of stent patency and obstruction-related outcomes within a practical experimental timeframe. Fig. 7a illustrates the overall flow of the rabbit experiment. Before the sacrifice, X-ray imaging was performed to evaluate stent patency. The contrast medium readily passed through both non-coated and ELFS-coated stents, confirming their initial patency (Fig. 7b). However, after two months, non-coated stent group exhibited complete obstruction, preventing the contrast medium from flowing through. In contrast, ELFS-coated stents maintained patency similar to their initial state. To further validate these findings, the liver and common bile duct were explanted, and a patency test was conducted using phosphate-buffered saline (PBS) dyed with purple ink (Fig. 7c). The solution was injected through a syringe into the stent region to assess fluid passage. In non-coated stent group, severe obstruction led to a complete overflow of the dyed solution, indicating a loss of patency. Conversely, in ELFS-coated stent group, the solution passed through the stent without restriction, demonstrating sustained patency over the two-month implantation period. During necropsy, non-coated stent group showed significant bile leakage in the abdomen, liver, and intestine due to its obstruction (green dashed outline). In contrast, no bile leakage was detected in rabbits implanted with ELFS-coated stents, demonstrating the coating's ability to maintain bile flow and prevent leakage-related damage. Additionally, in non-coated stent group, the gallbladder exhibited a more than seven-fold increase in size compared to its pre-surgical state (red dashed outline). Moreover, the liver demonstrated a two-fold enlargement, and the common bile duct was notably dilated (blue dashed outline), suggesting significant bile retention and hepatobiliary stress. However, in ELFS-coated stent group, no visible damage to the gallbladder, liver, or common bile duct was observed upon macroscopic examination. This indicates that ELFS coating effectively prevented bile accumulation and obstruction-induced hepatobiliary complications (Fig. 7d). Optical images of the retrieved stents clearly reveal marked differences between groups: non-coated stent showed complete luminal occlusion, whereas ELFS-coated stent maintained an open lumen (Fig. 7e). To further analyze the surface condition, the retrieved stents were longitudinally cut for SEM imaging. Non-coated stent exhibited substantial bile accumulation, visualized by green pseudo-coloring, which indicates significant biofouling. In contrast, ELFS-coated stent exhibited a clean surface with no bile adhesion, demonstrating its superior anti-fouling properties (Fig. 7f). These findings further confirm the effectiveness of ELFS in preventing bile-induced occlusion and maintaining stent patency. Blood tests were performed to evaluate liver function by measuring bilirubin, ALT, and AST. In non-coated stent group, bilirubin levels rose significantly after four weeks, indicating biliary obstruction and impaired bile excretion typically associated with cholestasis or bile duct occlusion. Persistent bile accumulation can lead to hepatocellular damage, inflammation, and subsequent liver dysfunction. In addition, after two months, ALT and AST levels showed a marked increase in non-coated stent group, indicating significant liver stress and potential hepatocellular injury. Serum ALT levels were elevated in non-coated stent group, indicating hepatocellular damage. Notably, AST levels also increased, and the disproportionate rise relative to ALT suggests progressive impairment of hepatobiliary function and potential fibrotic changes. These findings strongly indicate that bile duct obstruction caused by stent occlusion in non-coated group contributed to progressive hepatic impairment over time. In contrast, ELFS-coated stent group exhibited relatively stable bilirubin, ALT, and AST levels, suggesting that bile flow was maintained, preventing liver damage (Fig. 7g). These results further reinforce the role of ELFS in preserving bile duct patency and reducing the risk of bile-induced hepatotoxicity. We analyzed the bile duct to assess structural changes following stent implantation. Optical imaging revealed that the bile duct in non-coated stent group exhibited approximately a seven-fold dilation compared to its initial state due to prolonged obstruction. To further investigate these structural alterations, histological analysis was performed. The results confirmed fibrosis progression around the stent implantation site, suggesting a chronic inflammatory response and tissue remodeling due to bile retention. Additionally, muscular hypertrophy was observed in the bile duct wall. This phenomenon is likely a compensatory response triggered by prolonged bile outflow obstruction, leading to increased smooth muscle thickness to overcome the resistance to bile drainage. In contrast, ELFS-coated stent group exhibited minimal bile duct dilation with no significant fibrosis or muscular hypertrophy. These findings suggest that bile flow was maintained, thereby preventing pathological tissue remodeling (Fig. 7h and Fig. S5b). Next, we examined the liver to evaluate structural and pathological changes. As observed in the optical images, non-coated stent group exhibited greenish discoloration of the liver. This finding indicates significant bile accumulation resulting from impaired bile excretion (Fig. 7i). This bile retention is a hallmark of cholestasis, which can lead to hepatic injury and progressive fibrosis over time. Histological analysis further revealed severe hepatic fibrosis in non-coated stent group, as evidenced by MT staining. Hepatic fibrosis is primarily caused by chronic bile duct obstruction, which induces prolonged inflammation, hepatocellular damage, and activation of hepatic stellate cells. These activated stellate cells are known to produce excessive ECM proteins, leading to the development of fibrotic tissue and potential progression to secondary biliary cirrhosis. In contrast, ELFS-coated stent group did not show signs of bile accumulation, liver discoloration, or severe fibrosis, suggesting that bile flow was maintained, preventing hepatobiliary complications. Next, we analyzed the condition of the bile (Fig. S5c). As observed in the optical images, the bile from non-coated stent group became cloudy green. Viscosity measurements revealed a significant increase in non-coated group, due to bile stasis, mucin hypersecretion, accumulation of cellular debris, and bacteria (Fig. S5d). In contrast, ELFS-coated stent group maintained bile clarity and normal viscosity. These findings suggest that bile flow was preserved, thereby preventing pathological alterations in bile composition. CAs and sliding angles (SA) analysis were consistent with viscosity results. Non-coated group demonstrated reduced CA and SA, due to bile stasis and pathological changes led to a reduction in viscosity and surface tension (Fig. S5e and f). To further examine surface-associated changes after 2-month implantation, SEM– energy dispersive X-ray spectroscopy (EDS) elemental mapping was performed on explanted stents (Fig. S6). Non-coated stents exhibited irregular aggregated debris accompanied by a pronounced nitrogen (N) signal, consistent with the accumulation of biologically derived organic deposits under bile stasis conditions. In contrast, ELFS-coated stents showed minimal N signal and a clear fluorine (F) distribution, indicating reduced biological fouling and the sustained presence of fluorinated surface characteristics. Carbon (C) signals were observed in both groups, corresponding to the polyethylene stent substrate. Notably, the retained fluorine (F) signal observed on explanted ELFS-coated stents is more likely attributed to the underlying PFP scaffold rather than the fluorinated lubricant itself. As shown in the *in vitro* bile-flow study (Fig. 3e), the fluorinated lubricant is gradually depleted under sustained bile shear, suggesting that its contribution to anti-fouling behavior is most prominent during the early implantation phase. Despite this gradual lubricant loss, the persistent presence of fluorinated surface features indicates that the PFP scaffold remains stably associated with the stent surface, enabling continued resistance to biological adhesion at later stages of implantation.

## 3 Conclusion

This study demonstrates that ELFS is a practical durable and clinically compatible anti-fouling coating for biliary stents that addresses biofouling and FBR associated occlusion. ELFS effectively prevented biofilm and bile sludge accumulation and suppressed adverse host immune responses. These combined effects resulted in a significant prolongation of stent patency in a rabbit common bile duct model with stable long-term performance under bile-rich conditions. Importantly, ELFS enables a simple, conformal surface modification of commercially available plastic stents without requiring complex micro- or nanostructures. This feature allows uniform coating of long and tubular geometries and ensures compatibility with clinically relevant materials and standard sterilization procedures. Mechanistically, the sustained stent patency arises from a stepwise antifouling strategy, in which the lubricant-infused ELFS interface dominates early-stage antifouling and slippery behavior under bile flow, while the underlying PFP scaffold remains stably associated with the stent surface and continues to provide baseline anti-adhesive properties at later stages. The sustained stent patency observed in the rabbit common bile duct model further highlights the potential of ELFS to mitigate biliary obstruction and reduce downstream complications associated with stent failure. Overall, this work establishes ELFS as a robust and practical surface modification strategy for improving the long-term functionality of biliary stents. These findings provide a foundation for future translational efforts aimed at applying antifouling coatings to implantable medical devices exposed to challenging biological environments.

## 4 Experimental section

### 4.1 ELFS fabrication

Dopamine hydrochloride was obtained from Sigma-Aldrich, USA, while Tris buffer solution and H_2_O_2_ solution were sourced from Thermo Fisher Scientific, USA. CuSO_4_ was acquired from Duksan Company, South Korea. The fluorinated polymer precursor CTX-109AE and its corresponding solvent, CT-Solv. 100E, were supplied by AGC Chemical Inc. (Japan) and were used as the perfluoropolymer (PFP) scaffold material throughout this study. Perfluoropolyether **(**PFPE, Krytox GPL 101**),** used as the infused lubricant, was purchased from Chemours. For PDA coating deposition, a polydopamine solution was prepared by dissolving dopamine hydrochloride (2 mg/mL) in a Tris buffer solution (pH 8.5, 50 mM) purchased from Thermo Fisher Scientific, USA. Oxidizing agents, CuSO_4_ (5 mM) and H_2_O_2_ (19.6 mM), were then introduced into the solution. The substrates were submerged in the polydopamine solution under ambient conditions for at least 20 min. Following deposition, the PDA-coated samples underwent sonication in deionized water for 90 s and were subsequently dried in an oven at 60 °C for 2 h. Next, the PFP coating material (CTX-109AE, 9%) was diluted to a 2% concentration using CT-Solv. 100E as the solvent. The PDA-coated samples were immersed in the PFP coating solution and then air-dried at room temperature for 5 min. To facilitate amide bond formation during the curing process, the substrates were thermally cured in an oven at 100 °C for 4 h. After curing, the PFP-coated samples were immersed in PFPE liquid **(**PFPE, Krytox GPL 101**)** for 10 min to create a lubricant-infused layer. Any excess liquid was removed by positioning the samples at a 45° angle for 15 min before use. Prior to application, PFPE was filtered using a 0.2 μm syringe filter to eliminate any unwanted particles. Unless otherwise specified, the same procedure was followed for all materials mentioned above.

### 4.2 Characterization of the ELFS coating

To assess the surface chemical composition, X-ray photoelectron spectroscopy (XPS, PHI 5000 VersaProbe, ULVAC PHI) was employed. The XPS system was equipped with an Al K-alpha X-ray source featuring a spot size of 100 μm^2^ s. Additionally, contact angle (CA) and sliding angle (SA) measurements under static conditions were performed on both non-coated and ELFS coated polyethylene substrates using a CA measurement system equipped with a dynamic image capture camera (SmartDrop Standard Plus, FEMTOBIOMED Inc.). To determine the CA values, 10 μL droplets of various liquids, including bile, FBS, defibrinated horse blood (Kisan Bio), dimethyl sulfoxide (DMSO), ethylene glycol (EG) (Sigma-Aldrich), were dispensed onto the substrate surface. SA values were obtained by tilting the substrate at a rate of one degree per second and recording the angle at which the droplets began to roll off. The thickness of the PDA and PDA–PFP coatings was measured using a surface profilometer (Dektak, Bruker). For thickness measurement, a defined region of the coating was selectively masked prior to coating to create a step edge between coated and non-coated areas. Profilometry scans were then performed across this step edge, and the coating thickness was determined from the height difference between the coated and non-coated regions. Multiple measurements were conducted at different locations on each sample to ensure reproducibility, and the averaged values were reported.

### 4.3 Anti-fouling characteristics

Alexa Fluor 488-conjugated albumin from bovine serum (BSA; A13100, Invitrogen) and fibrinogen from human plasma (F13191, Invitrogen) were used. To evaluate the anti-adhesive properties of the ELFS coating against plasma proteins, both non-coated and ELFS-coated PDMS substrates were immersed in 5 mL of aqueous protein solutions (1 mg/mL) and incubated at 37 °C for 24 h. After incubation, the samples were thoroughly rinsed with deionized water and allowed to air-dry under ambient conditions. Fluorescence images were acquired using an inverted fluorescence microscope (IX81, Olympus, Japan) under identical imaging settings for all samples. Quantitative analysis of protein adsorption was performed using ImageJ/FIJI software. Fluorescence intensity was quantified after background subtraction and normalized to the surface area of the substrate. For each condition, multiple regions of interest were analyzed per sample, and the values were averaged across independent samples (n = 4). For PDMS substrate preparation, 10 g of PDMS was mixed with a base polymer and curing agent in a 10:1 (w/w) ratio. The mixture was degassed in a vacuum chamber to remove air bubbles and carefully poured into a square polystyrene Petri dish (140 mm in diameter). The PDMS was then cured in an oven at 70 °C for 2 h. The blood fouling test was conducted using defibrinated horse blood (Kisanbio, Korea) on both non-coated and ELFS coated polyethylene tubing. The horse blood was constantly infused into the tubing using a syringe pump.

### 4.4 Mechanical stability test

The mechanical durability of ELFS was evaluated using the cross-cut adhesion test with glass substrate, following the ISO 2409 standard. This assessment involved creating a grid pattern on the coated samples by making two sets of six cuts, each spaced 1 mm apart, perpendicular to one another. This process resulted in a matrix of 25 small blocks on the surface. The test was conducted on PEG-based anti-fouling coatings as a negative control and ELFS coated glass substrates. After the grid was created, adhesive tape was applied to the surface and subsequently peeled off at a 90° angle. This tape-peeling process was repeated ten times to assess adhesion durability. Furthermore, to evaluate the coating's stability in an aqueous environment, the fouling resistance of the ELFS coating was examined before and after exposure to ultrasonication, water flushing, and water shearing treatments. For the ultrasonication test, the ELFS coated glass substrate was placed in a Petri dish and immersed in PBS. The dish was then transferred to an ultrasonication bath operating at 20 kHz without temperature control. In the water-flushing experiment, a peristaltic pump was used to circulate deionized water perpendicularly over the ELFS coated glass substrate at a flow rate of 5 mL/s. For the water shearing test, the ELFS coated glass substrate was positioned at the center of a beaker, submerged in PBS, and subjected to shear forces generated by a magnetic stirring bar rotating at 1000 rpm. Following these durability tests, the substrates were incubated in a suspension of humic acid in PBS solution (5 mg/mL) for one day. The degree of fouling was then assessed through UV-Vis spectrophotometry, measuring absorbance in the range of 200 to 254 nm.

### 4.5 Stability under constant flow condition

Lubricant retention on a macroscale level was evaluated under varying constant bile flow conditions through microchannels at predetermined time intervals. To enable visualization, the lubricant was stained with a red fluorescent dye. The amount of remaining lubricant within the microchannels of the PreD chip was analyzed using an inverted fluorescence microscope (IX81, Olympus, Japan) and quantified with the ImageJ/FIJI software.

### 4.6 Self-healing of EFLS

ELFS-coated glass substrates were prepared by applying a 3% PFP coating material. To evaluate the heat-mediated self-healing properties of ELFS, a mechanical scratch was introduced using a sharp knife. The damaged substrate was then immersed in lubricant for at least 4 h to allow sufficient swelling, followed by heating on a hot plate at 100 °C. Optical images were captured at 0, 2, 12, and 24 h, and the percentage of the damaged area was quantified using the ImageJ/FIJI software.

### 4.7 Swab abrasion test

Non-coated and ELFS coated glass substrates were prepared following the previously described method. The samples were secured using a clip, and each surface was manually rubbed in a horizontal direction with a pure cotton swab (Daihan Scientific Co.). To evaluate the self-healing capability of the substrates, water droplets were applied before and after the abrasion, allowing for a comparative analysis of surface recovery.

### 4.8 Sterilization resistance test

To evaluate the sterilization resistance of robust ELFS, various sterilization methods, including autoclave, ethylene oxide (EO) sterilization, and gamma sterilization, were performed. All sterilization procedures were applied to both non-coated and ELFS-coated PE based plastic stent. Autoclave sterilization was conducted for 3, 6, and 9 cycles on each sample. EO sterilization was carried out by Cobuild Inc., while gamma sterilization was performed at a dosage of 25 kGy. After EO and gamma sterilization, the samples were exposed to air for stabilization. For plasma treatment under harsh conditions, the samples were subjected to 50 SCCM oxygen plasma at 100W for 2 min, followed by air exposure to allow for chemical stabilization.

### 4.9 Oxidation degradation test

PDA-coated and ELFS-coated glass substrate were prepared following the previously described method. The PDA-coated glass substrate or material is first rinsed with deionized water (DI Water) to remove any impurities. After thorough washing, the sample is dried completely before use to ensure consistency in the experimental conditions. Next, the FeCl_3_ + H_2_O_2_ reaction solution is prepared. A 1 mM FeCl_3_ (Sigma-Aldrich, 157740) solution is obtained by dissolving 0.027 g of FeCl_3_·6H_2_O in 100 mL of DI Water, and the concentration can be adjusted between 1 mM as needed. The H_2_O_2_ solution (10%) is prepared by diluting 30% H_2_O_2_ with DI Water. The pH of the solution is adjusted to 3 using 1 M HCl, as this pH range facilitates the Fe^3+^ to Fe^2+^ transition, which enhances the oxidative reaction. For the PDA degradation process, the prepared PDA-coated sample is immersed in the FeCl_3_ + H_2_O_2_ solution. The reaction time is monitored, ranging from 1 to 7 days. The color fading of the PDA coating is noted visually, and UV-Vis spectrophotometry is used to quantify the degradation process by tracking the decrease in the characteristic absorbance peak (∼350–400 nm). After the reaction, the sample undergoes post-treatment washing to remove residual chemicals. The sample is rinsed at least three times with DI Water, followed by immersion in a neutral buffer solution (DI Water or NaHCO_3_ buffer, pH 7–8) for 10 min to eliminate any remaining oxidizing agents. Once washed, the sample is completely dried before further analysis.

### 4.10 *In vitro* biocompatibility test

The non-coated and ELFS-coated PE based plastic stent fragments were prepared, following a previously reported method. To assess cell viability and morphological changes, a two-chamber Transwell system (8 μm pore size; Corning) was utilized. The prepared stents were placed on the Transwell insert, and NIH-3T3 fibroblasts (ATCC CRL-1658; 0.5 × 10^5^ cells mL^−1^) or human biliary epithelial SNU-1079 cells (Korean Cell Line Bank, KCLB No. 01079; 0.5 × 10^5^ cells mL^−1^) were cultured in 2 mL of DMEM supplemented with 10% bovine calf serum and 1% penicillin–streptomycin. Following the manufacturer's protocol, cell viability was analyzed using a Live/Dead kit (L3224, Invitrogen, USA). Fluorescence images were captured at 10 × magnification using an inverted fluorescence microscope (IX81, Olympus, Japan). Similarly, morphological changes were assessed by calculating the aspect ratio, defined as the ratio of the major cell axis to the minor axis. To visualize the actin cytoskeleton, fibroblast cells were stained with Alexa 594-conjugated phalloidin (Thermo Scientific, Pittsburgh, PA, USA). The fibroblast morphology was observed using a confocal microscope (LSM 980, Carl Zeiss, Oberkochen, Germany) and quantified using the ImageJ/FIJI software program.

### 4.11 Selective cell adhesion test

Various shapes of ELFS coating, including heart, triangle, square, and cross, was applied to cell culture plates through a syringe-based coating method. Subsequently, two types of cells (IH 3T3, RAW 264.7) were cultured within 2 mL of DMEM supplemented with 10% bovine calf serum and 1% penicillin-streptomycin. Following the manufacturer's protocol, the cell was stained using a Live/Dead kit (L3224, Invitrogen, USA). Fluorescence images were captured at 10 × magnification using an inverted fluorescence microscope (IX81, Olympus, Japan). Cell adhesion and patterning were quantitatively analyzed from fluorescence microscopy images using ImageJ (NIH). All images were acquired under identical microscope settings (objective, exposure time, gain, and illumination intensity) without signal saturation. For each sample, regions of interest (ROIs) corresponding to ELFS-coated patterned areas and adjacent non-coated regions were manually defined, excluding boundary regions to avoid edge effects. Background fluorescence was subtracted using the rolling ball algorithm, and images were subsequently thresholded using an identical thresholding criterion applied consistently across all samples. Cell-covered area fraction (%) was calculated as the ratio of thresholded cell-positive area to the total ROI area. In parallel, integrated fluorescence intensity was measured within each ROI and normalized to surface area to obtain fluorescence intensity per unit area. Quantitative data were obtained from three independent experiments, with at least five randomly selected fields of view analyzed per condition in each experiment. For statistical analysis, values from multiple fields within each experiment were averaged to yield a single biological replicate. Data are presented as mean ± standard deviation, and statistical significance was assessed using a two-tailed Student's t-test unless otherwise noted.

### 4.12 Bacteria test

The antibacterial evaluation was conducted using *E. coli* (ATCC 8739) as a model gram-negative bacterium and *S. aureus* (ATCC 6538) as a model gram-positive bacterium, both provided by the Korean Agricultural Culture Collection (Jeonbuk, Korea). The bacterial strains were cultured in sterilized Luria-Bertani (LB) broth and incubated in a temperature-controlled shaker at 37 °C with a rotation speed of 100 rpm for 12 h. The bacterial suspension was then diluted to a final concentration of 10^5^ cells/mL in LB broth and dispensed onto the surface of an agar plate. The samples were incubated at 37 °C for 24 h, allowing biofilm formation during bacterial proliferation.

### 4.13 *In vitro* biofilm formation test

To begin, a bacterial strain such as *Staphylococcus aureus* and *Escherichia coli* is selected. A single colony is picked from an agar plate and inoculated into 5 mL of an LB broth. The culture is incubated at 37 °C with shaking at 150–200 rpm for 12 to 18 h to ensure optimal bacterial growth. After incubation, the bacterial culture is diluted to an OD600 of 0.05–0.1, which corresponds to approximately 1 × 10^6^ CFU/mL, using fresh broth to standardize the initial bacterial concentration. For biofilm formation, the non-coated and ELFS coated stent fragments are sterilized by autoclaving. The sterilized substrate is then placed in a 24-well or 6-well plate using sterile forceps. To initiate biofilm formation, 2 mL of the diluted bacterial suspension is added to each well containing the substrate. A negative control is also prepared by adding only sterile broth without bacteria to ensure the absence of contamination. The plate is incubated at 37 °C under static conditions for 24 h, allowing biofilms to develop on the substrate surface. After incubation, the planktonic (non-adherent) bacteria are carefully removed by aspirating the medium, and each well is washed three times with 2 mL of sterile phosphate-buffered saline (PBS) to remove loosely attached cells. Biofilm-like accumulation was induced onto PE plastic stent substrates (non-coated and ELFS-coated) were cut into (1 × 1 cm^2^) identical dimensions and sterilized by sequential immersion in 70% ethanol for 30 min, followed by ultraviolet (UV) irradiation for 30 min on each side. The sterilized substrates were then placed in sterile 24-well culture plates. Bacterial cultures were grown overnight in appropriate culture medium at 37 °C with shaking, diluted to an optical density at 600 nm (OD_600_) of approximately 0.1, and added to each well to fully immerse the PE substrates. Samples were incubated statically at 37 °C to promote bacterial adhesion and biofilm formation. After the initial adhesion phase (24 h), the culture medium was gently replaced to remove non-adherent bacteria. Biofilms were allowed to mature for 3–5 days under static conditions, with fresh medium supplied every 24 h. Following incubation, the substrates were gently rinsed with phosphate-buffered saline (PBS) to remove loosely attached bacteria. To analyze biofilm-like accumulation and morphology, samples were fixed in 2.5% glutaraldehyde for 2 h at 4 °C, followed by sequential dehydration in graded ethanol solutions (30%, 50%, 70%, 90%, and 100%) for 15 min each. The dehydrated samples were dried under vacuum, sputter-coated with palladium, and examined using a field-emission scanning electron microscope (FE-SEM; JEOL, JSM-IT500HR).

### 4.14 *In vivo* imaging of immune cell dynamics using dorsal skinfold chamber implantation

Female C57BL/6 mice (8 weeks old) were obtained from Orient Bio Inc. (Gyeonggi-do, South Korea) and housed under semispecific pathogen-free (semi-SPF) conditions with a 12-h light/dark cycle for quarantine and acclimatization. To fluorescently label immune cells, F4/80 antibody (IVIM, IVITM-991-0054, IVIM technology, South Korea, diluted 1:4 in PBS) and Ly6G antibody (IVIM, IVITM-991-0024, IVIM technology, South Korea, diluted 1:2) were intravenously injected (100 μL each) via the tail vein 18 h prior to imaging for macrophage and neutrophil targeting, respectively. CD31 antibody (IVIM, IVITM-991-0024, IVIM technology, South Korea, diluted 1:2) was additionally injected (100 μL) 1 h before imaging to visualize vascular structures. Mice were anesthetized with an intramuscular injection (100 μL) of Zoletil 50, Rompun, and PBS mixed at a ratio of 1:1:6, followed by dorsal hair removal and disinfection. A dorsal skinfold chamber (DSC; IVIM) was surgically installed, and polyethylene film with 0.1 mm height and a diameter of 0.5 cm with or without ELFS coating was implanted intradermally before chamber closure. Intravital imaging was performed at 0, 3, 24, and 72 h post-implantation using a confocal intravital microscope (IVIM, IVM-CM3, IVIM technology, South Korea) under continuous monitoring of anesthetic depth and respiration. Fluorescence signals were acquired with channel settings of GFP 10, RFP 40, and Cy5 20, and 9–10 s video sequences were recorded for each time point. Raw image data were processed using the manufacturer-provided AI-image Denoiser (IVIM) to reduce optical noise, and subsequent analyses were conducted with IVIM Studio software to quantify immune cell infiltration and visualize temporal changes in cell distribution around the implant. Quantitative analysis of immune cell dynamics was performed by counting fluorescently labeled neutrophils (Ly6G^+^) and macrophages (F4/80^+^) within a defined region of interest (ROI) surrounding the implanted sample. For each mouse, multiple imaging fields were analyzed at each time point, and the values were averaged to obtain a representative measurement per animal. Data were then pooled across animals within each group for statistical comparison.

### 4.15 Biocompatibility with mouse model

Female ICR mice (7 weeks old) were obtained from Orient Bio Inc. (Gyeonggi-do, South Korea) and housed under semispecific pathogen-free (semi-SPF) conditions with a 12-h light/dark cycle for quarantine and acclimatization. Non-coated and ELFS coated stent fragments were prepared following previously established methods. Each sample was then implanted subcutaneously into left side of the back of female ICR mice. Prior to implantation, the mice were anesthetized via intramuscular injection of ketamine (100 mg/kg; Yuhan, Seoul, Korea) and xylazine (20 mg/kg; Bayer Korea, Ansan, Korea), followed by shaving of the surgical area. A longitudinal incision of approximately 15 mm was made on the skin using surgical scissors, and subcutaneous pockets were created on either side of the incision using blunt forceps to accommodate the hydrogel disks. After implantation, the incisions were closed with surgical sutures.

### 4.16 Biofilm formation confirmation with mouse model

*In vivo* biofilm formation on implanted PE based plastic stent fragments was evaluated using female ICR mice. To optimize the gallbladder environment for stent implantation, the cystic duct was surgically clipped prior to the experiment. This procedure was performed to induce gallbladder enlargement, creating a suitable space for the implantation of the prepared stent fragments. The stent fragments were prepared as circular disk-shaped samples using a 0.5 mm biopsy punch, resulting in a uniform diameter of 0.5 mm and a planar surface area of approximately 0.20 mm^2^ per fragment. Following the cystic duct clipping, the prepared stent fragments were carefully implanted into the gallbladder of the mice under aseptic surgical conditions. The implantation procedure was conducted to assess the extent of biofilm formation on the stent surface over time under physiological conditions. The mice were then maintained under standard housing conditions with free access to food and water to allow for normal post-surgical recovery. To evaluate biofilm formation on the implanted stents, the mice were sacrificed at two different time points, specifically after one week and six months post-implantation. The time intervals were selected to observe both early-stage biofilm development (short-term, one week) and long-term biofilm maturation (six months). Upon sacrifice, the gallbladder and surrounding tissues were carefully excised, and the implanted stent fragments were retrieved for further analysis.

### 4.17 Histological analysis (mouse)

After experiments, the mouse were euthanized, and the surrounding tissue along with the stent fragments was extracted and collected. The explanted samples were fixed in 4% paraformaldehyde (PFA) overnight at room temperature and subsequently embedded in paraffin wax. Thin sections, measuring 3 to 5 μm in thickness, were prepared and mounted onto slides for histological staining. To assess the inflammatory response, tissue sections were stained with H&E, where cell nuclei appeared blue, and the cytoplasm was stained pink. Collagen deposition and organization were analyzed using Masson's trichrome staining, which colors collagen blue, cytoplasm red, and nuclei dark red or purple. Bright-field images of the stained samples were captured using an inverted fluorescence microscope (IX81, Olympus, Japan). To identify mast cells at one-week post-implantation, toluidine blue staining begins with the fixation of tissue sections in 4% paraformaldehyde or another appropriate fixative for at least 30 min at room temperature. The slides undergo deparaffinization by immersion in xylene for 5 min, followed by rehydration through a graded ethanol series (100%, 95%, 70%) and a final rinse in distilled water. The slides are then stained in a 0.1% toluidine blue solution, prepared in 0.1 M acetate buffer at pH 4.0–4.5, for 2–5 min at room temperature. After staining, the slides are briefly rinsed in distilled water or 0.1 M acetate buffer to remove excess dye. If differentiation is needed to reduce background staining, a quick dip in 95% ethanol can be performed. For permanent mounting, the slides are dehydrated through graded ethanol solutions (70%, 95%, 100%), followed by xylene. Finally, the slides are mounted with a permanent mounting medium and examined under a light microscope.

### 4.18 Bile duct stents patency test (Rabbit)

This experimental study was conducted using eight healthy SPF New Zealand white rabbits, obtained from Dooyeol Biotech Co., Ltd., Seoul, Republic of Korea. The rabbits were housed individually in stainless-steel cages within a controlled environment, where they underwent a 2-week acclimatization period at the Veterinary Medical Teaching Hospital of Chungnam National University, receiving appropriate care and nutrition. The study was approved by the Institutional Animal Care and Use Committee of Chungnam National University (permit number 202410A-CNU-235), and all procedures were carried out in accordance with established guidelines. All surgeries were performed under general anesthesia to minimize pain and discomfort. Following the experiments, humane euthanasia was performed on the scheduled date under general anesthesia via intravenous injection of a 10% potassium chloride solution.

All animals in this study underwent the same surgical procedure performed by a single surgeon, with the exception of the biliary stent used (non-coated stent, n = 4; ELFS-coated stent, n = 4). Each rabbit was preoxygenated with 100% oxygen using a flow-by technique. General anesthesia was induced with intravenous alfaxalone (3 mg/kg, IV; Alfaxan multidose; Jurox Pty. Limited, AUS), followed by endotracheal intubation. Anesthesia was maintained with 1.5–2.0% isoflurane (Ifran; Hana, ROK) in 100% oxygen. Perioperative analgesia was provided with a subcutaneous injection of meloxicam (0.6 mg/kg; Metacam Inj., Boehringer Ingelheim, Germany). Local infiltration at the abdominal incision site was achieved with a mixture of lidocaine (0.2 mg/kg, SC; Lidocaine Inj.; Jeil, ROK) and bupivacaine (0.5 mg/kg, SC; Bupivacaine Inj.; Myungmoon, ROK).

Once an adequate depth of anesthesia was achieved, the rabbit was placed in dorsal recumbency. The surgical area was sterilized with a solution of 10% povidone-iodine and 70% ethyl alcohol. A midline celiotomy, extending from the xiphoid process to the umbilicus, was performed, and the abdominal cavity was explored to evaluate the condition of the liver, gallbladder (GB), extrahepatic biliary tracts, and upper gastrointestinal tract. The hepatoduodenal ligament was dissected to expose the common bile duct (CBD). Wet, sterile laparotomy sponges were used to maintain a moist environment during the placement of the biliary stent.

For placement of the PE based plastic stent (inner diameter: 1.0 mm; outer diameter: 1.5 mm; purchased from Taewoong Medical, South Korea), two Doyen intestinal forceps were applied to the pyloric antrum and the duodenum. A duodenotomy, approximately 10 mm in length, was made at the antimesenteric region, opposite to the opening of the CBD. After confirming the opening, an 18-gauge catheter without a stylet was inserted, followed by placement of a 2-0 PDS suture through the catheter. Once the catheter was temporarily removed, a 1 cm long stent was threaded through the suture, and the catheter was used as a pusher to position the biliary stent inside the CBD. To prevent migration, anchoring sutures using 8-0 polypropylene (Prolene; Ethicon Inc., USA) were placed at two points on the CBD. The GB was gently compressed to check for bile leakage. The duodenotomy site was closed with a simple interrupted pattern using 5-0 PDS (PDS Plus; Ethicon Inc., USA). Enteric leakage was evaluated by injecting normal saline through a 3 cc syringe with a 25-gauge needle (Fig. S5a).

Postoperatively, the surgical site was disinfected with 10% povidone-iodine, and dressing changes were performed daily until the sutures were removed. To manage pain and prevent surgical site infection, each rabbit received meloxicam (0.6 mg/kg, SC) and enrofloxacin (20 mg/kg, SC; Baytril; Bayer AG, Germany) once daily for 1 week. Animals were monitored daily after surgery and euthanized 2 months after stent implantation for endpoint analyses. The study duration was determined based on a preliminary pilot study, in which 2 out of 3 rabbits implanted with non-coated stents died during longer implantation periods. To minimize animal suffering and to comply with institutional animal care and use guidelines, the experimental period was therefore limited to 2 months. At the experimental endpoint, rabbits were maintained under deep general anesthesia, and euthanasia was performed by intravenous administration of potassium chloride in accordance with approved ethical guidelines.

### 4.19 Blood analysis (Rabbit)

Blood samples for complete blood cell counts (CBC) and serum biochemistry were collected on postoperative days 1, 3, 5, and 7, with further analyses conducted weekly until the time of euthanasia. The CBC was performed using an automatic hematology analyzer (ProCyte Dx Hematology Analyzer; IDEXX Laboratories Inc., USA), allowing for the assessment of postoperative anemia, changes in inflammatory markers, and variations in individual leukocyte counts. Serum biochemistry was analyzed using a clinical blood chemistry analyzer (Catalyst One Chemistry Analyzer; IDEXX Laboratories Inc., USA). These tests were performed to evaluate liver function, identify signs of cholestasis or jaundice, and detect potential postoperative bile leakage. Liver enzymes, including AST, ALT, ALP, GGT, and TB, were measured to assess the liver's condition.

### 4.20 Statistical analysis

All statistical analyses were performed using GraphPad Prism (GraphPad Software Inc., USA) and Origin (OriginLab Corporation). Data are presented as mean ± standard deviation (SD). Prior to parametric analysis, data distributions were evaluated for normality using the Shapiro–Wilk test. For comparisons between two independent groups, an unpaired two-tailed Student's t-test was applied. For experiments involving multiple groups or time-course measurements, ordinary two-way analysis of variance (ANOVA) followed by Tukey's multiple comparison test was used. Sample sizes (n) and the specific statistical tests applied for each dataset are indicated in the corresponding figure captions. Statistical significance was defined as ∗p < 0.05, ∗∗p < 0.01, ∗∗∗p < 0.001, and ∗∗∗∗p < 0.0001; ns denotes not significant.

## CRediT authorship contribution statement

**Tae Young Kim:** Writing – original draft, Visualization, Methodology, Investigation, Formal analysis, Conceptualization. **Won-Jong Lee:** Visualization, Methodology, Investigation, Formal analysis. **Yurim Lee:** Methodology, Investigation. **Seo Jung Kim:** Methodology, Investigation. **Sungjin Min:** Methodology, Investigation. **Seyong Chung:** Methodology, Investigation. **Soo A Kim:** Methodology, Investigation. **Keun-Young Yook:** Methodology, Investigation. **Chang-Hwan Moon:** Methodology, Investigation. **Yeontaek Lee:** Visualization. **Kijun Park:** Methodology. **Dae-Hyun Kim:** Writing – original draft, Supervision, Conceptualization. **Jungmok Seo:** Writing – original draft, Supervision, Conceptualization.

## Ethics approval and consent to participate

All animal experiments were performed in compliance with the Guide for the Care and Use of Laboratory Animals and were approved by the Institutional Animal Care and Use Committee (IACUC) of Korea University (authorization number: KOREA-2025-0095), and the Institutional Animal Care and Use Committee (IACUC) of Yonsei University (authorization number: IACUC-A-202310-1744-01).

## Data availability statement

All data supporting the findings of this study are available within the article and its Supplementary Materials.

## Declaration of competing interest

The authors declare that they have no known competing financial interests or personal relationships that could have appeared to influence the work reported in this paper.
