# Supplementary material for: Slippery dopamine–fluoropolymer hybrid surface for improving biliary stent longevity
Source: Bioact Mater. 2026 Feb 13;61:210–28. doi: 10.1016/j.bioactmat.2026.02.003 (PMC12924897; doi:10.1016/j.bioactmat.2026.02.003)
Supplement: Multimedia component 1 [file mmc1.docx]

*
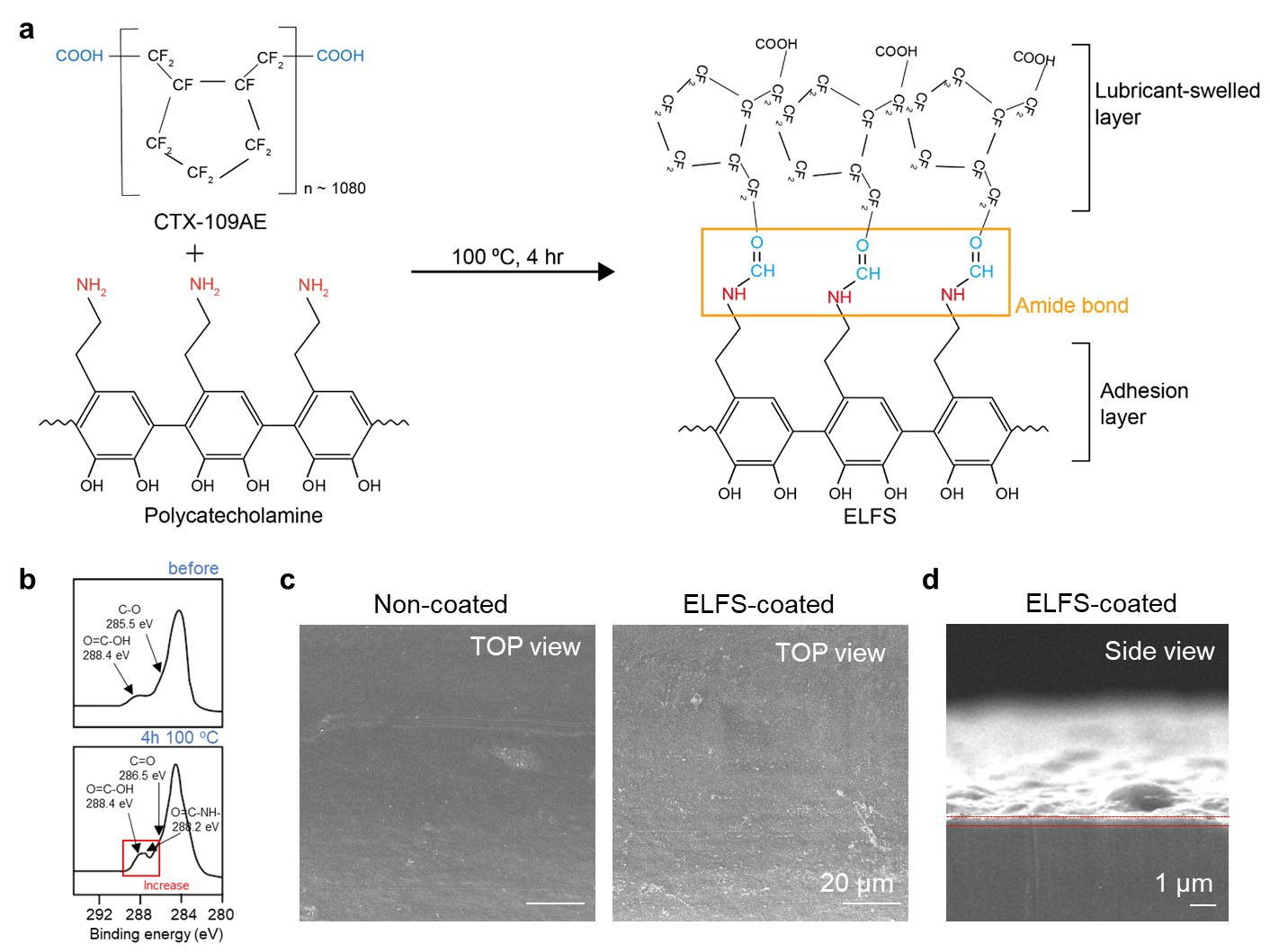
*

**Fig. S1.** (a) Schematic of a chemical reaction between CTX-109AE and polycatechoamine to form ELFS. (b) XPS analysis confirming heat-induced chemical changes in the coating after thermal curing (100 °C, 4 h). (c, d) Representative SEM images of non-coated and ELFS-coated stent fragments (n=3) (scale bars, 20 and1 µm, respectively).

**
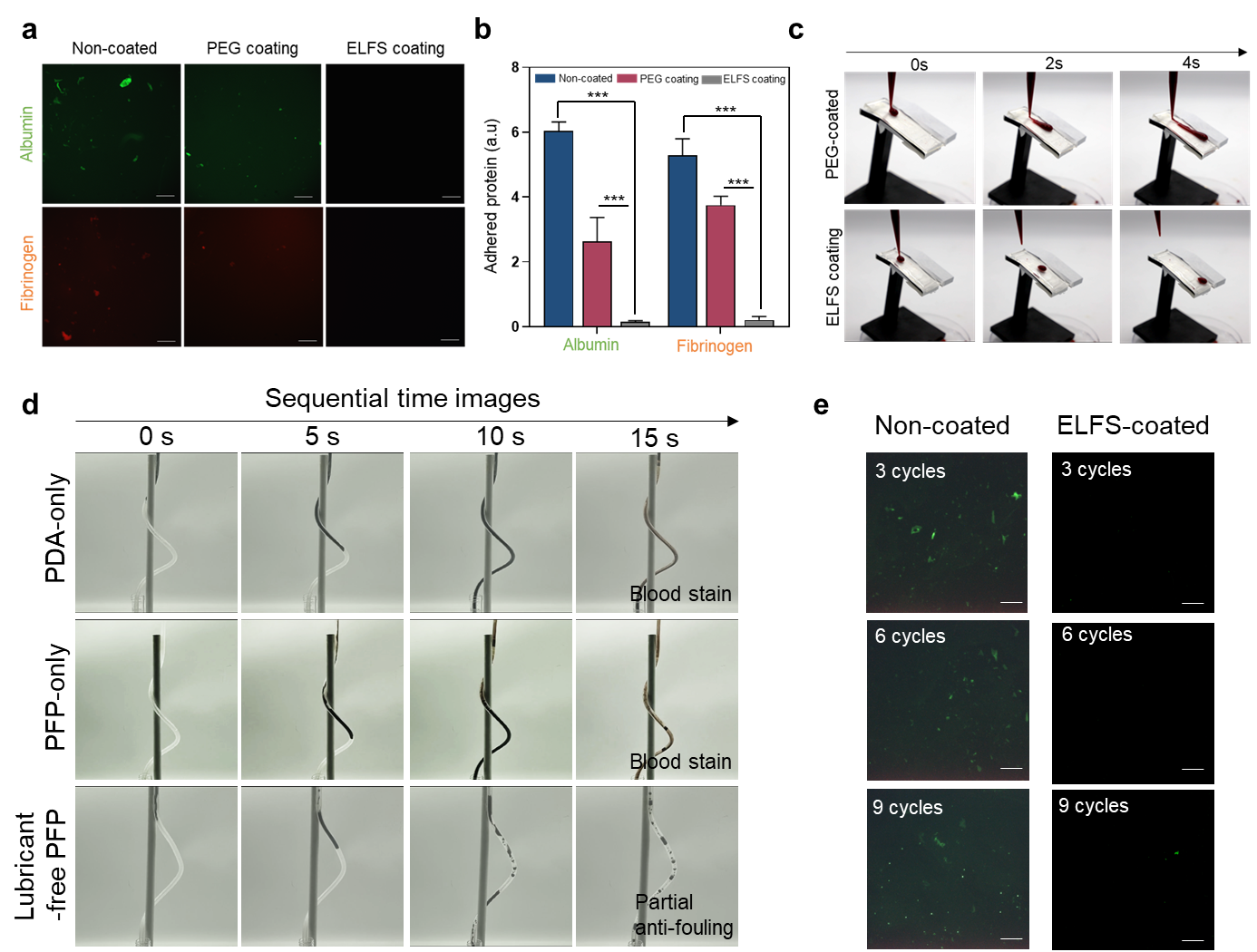
**

**Fig. S2.** (a, b) Representative fluorescence photographs of plasma proteins (albumin, fibrinogen) and their corresponding quantitative analysis (n=4). (c) Sequential optical photographs to confirm fouling resistance against horse blood (n=2). (d) Optical sequential photographs of blood staining experiment on PE medical tubing with PDA-only, PFP-only, and Lubricant-free PFP group. (e) Representative fluorescence photographs of albumin after autoclave sterilization (n=4). *(*P < 0.05,* ***P < 0.01, ***P < 0.001, and ****P < 0.0001). ns, not significant*


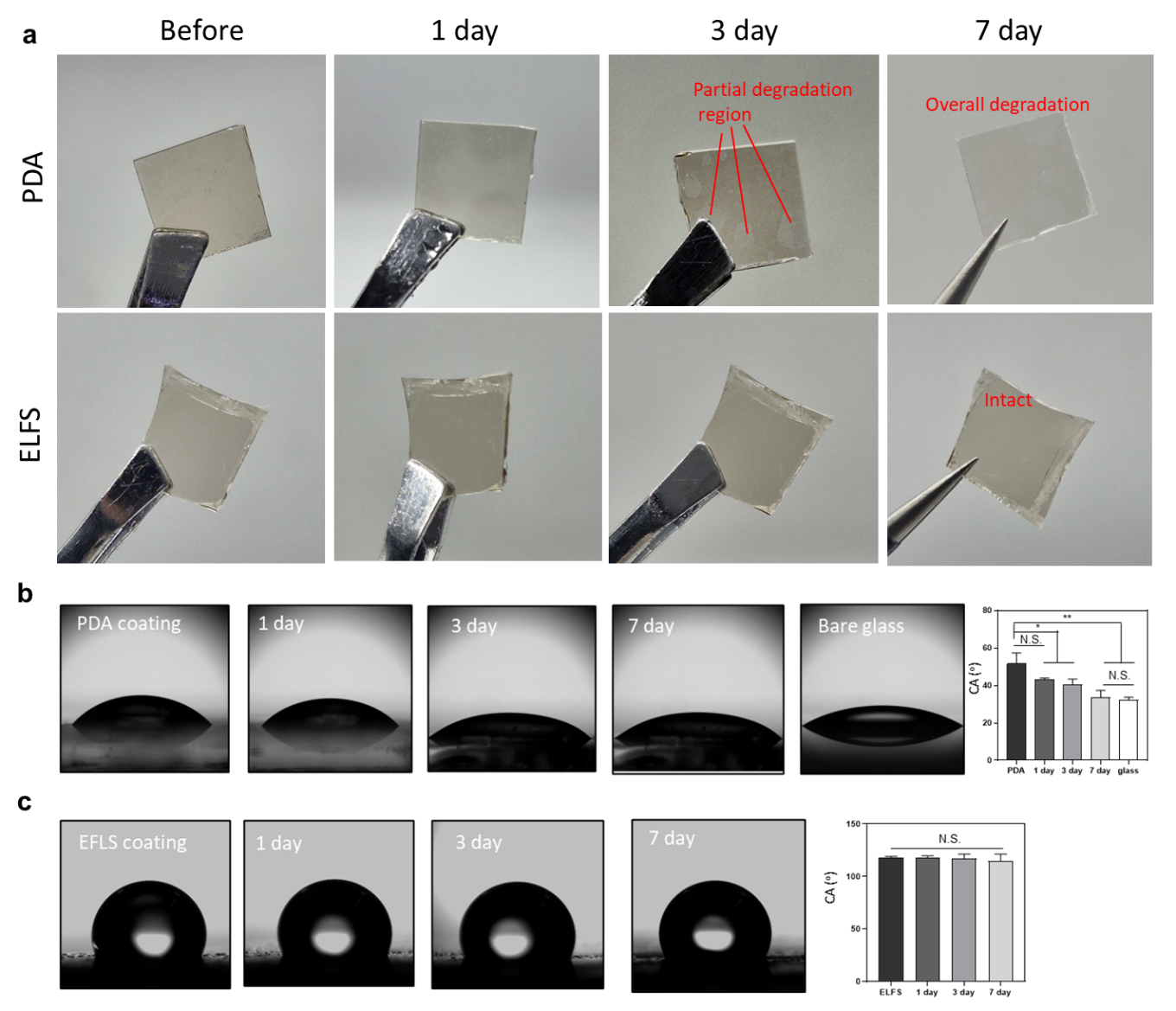


**Fig. S3.** (a) Representative time-lapse optical images of PDA and ELFS coated glass to evaluate degradation of the coatings under enzyme-derived oxidative environment (n=4). (b, c) Representative optical images of water droplet for measurements of CAs and its corresponding quantitative analysis (n=4). *(*P < 0.05,* ***P < 0.01, ***P < 0.001, and ****P < 0.0001). ns, not significant.*

**
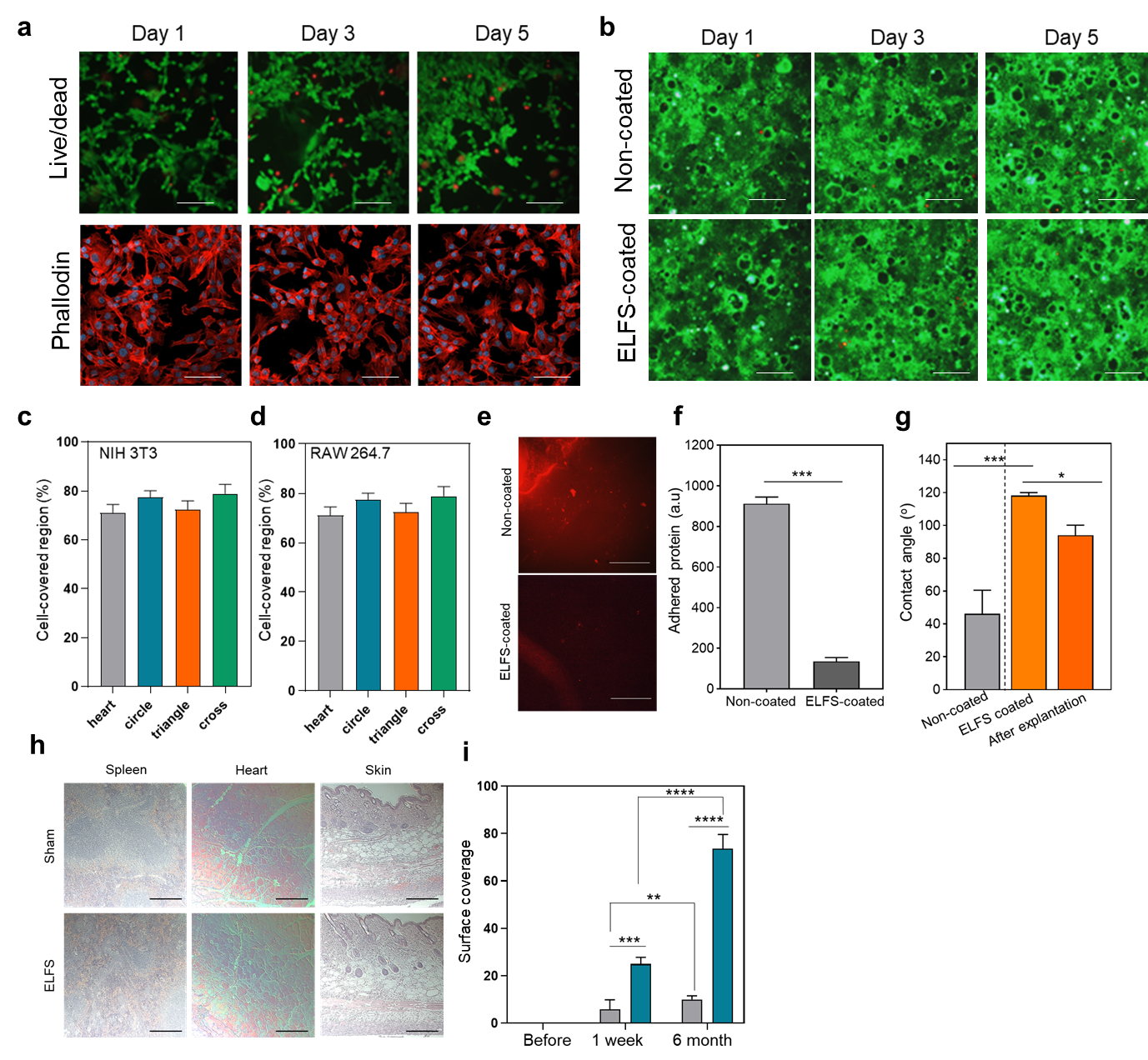
**

**Fig. S4.** (a, b) Fluorescence microscopic images of NIH 3T3 cells and human biliary epithelial cells stained with a live/dead kit and morphological analysis of NIH 3T3 cells stained for actin (red) and nucleus (blue), with fibroblast aspect ratio analysis (scale bars, 100 µm) (n=4). (c, d) Quantitative analysis of NIH 3T3 and RAW 264.7 cell patterning on ELFS-coated surfaces cell coverage (%) was quantified from fluorescence images by calculating the cell-positive area within defined regions of interest after background subtraction and thresholding (n = 4). (e, f) Fluorescence microscopy images of plasma protein (albumin) adhered on non-coated and ELFS coated stent fragments and its corresponding statistical analysis of the protein coverage (scale bars, 100 μm) (n = 3). (g) CAs measurements on the non-coated, ELFS coated stent fragments, and ELFS coated stent fragments after 1week implantation (n = 3). (h) H&E histological analysis of spleen, heart, and skin (scale bars, 100 µm) (n=4). (i) Quantification of biliary sludge surface coverage area of stent fragment (n=4). *(*P < 0.05,* ***P < 0.01, ***P < 0.001, and ****P < 0.0001). ns, not significant.*


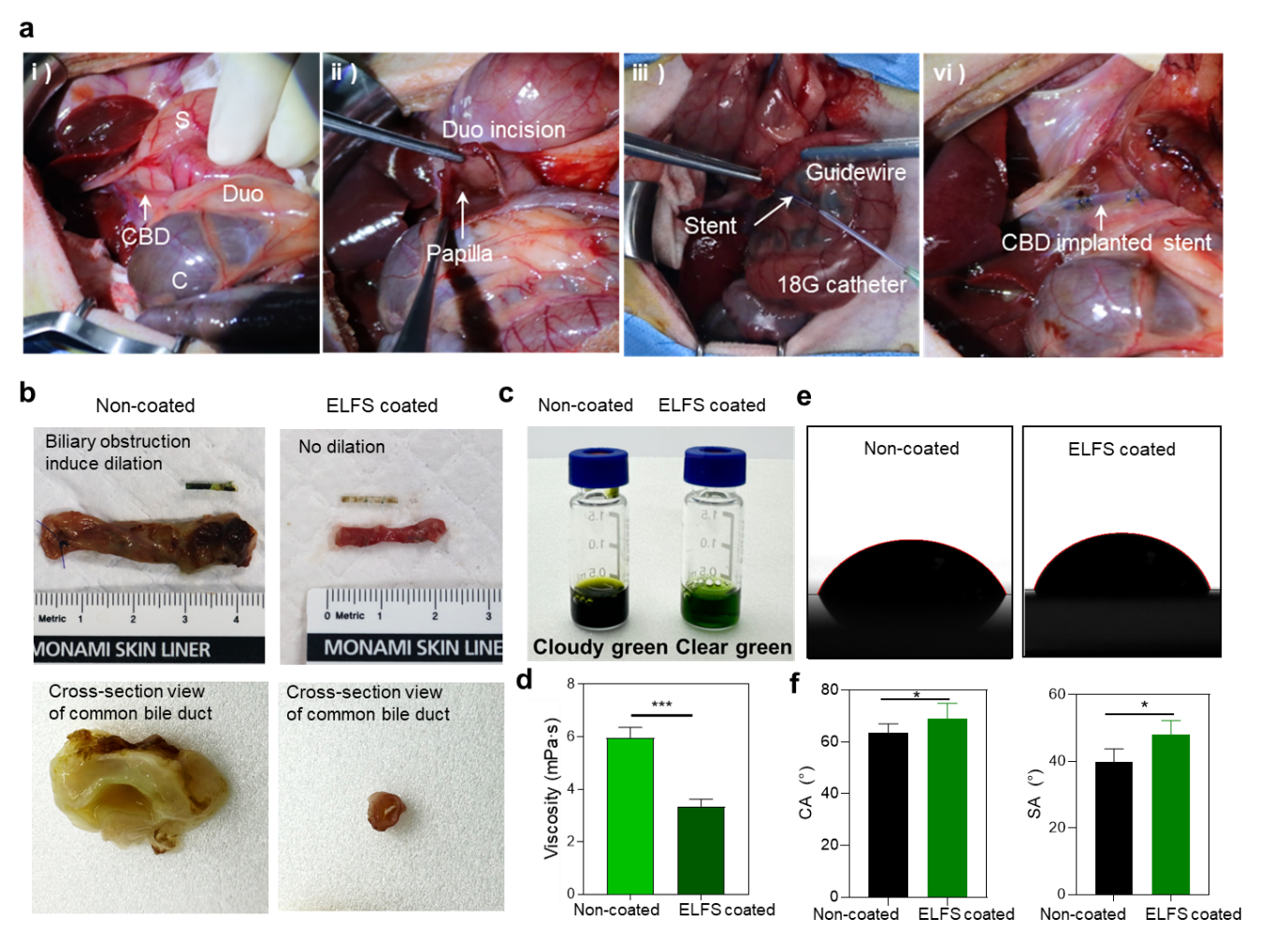


**Fig. S5.** (a) Optical photographs of the stent placement procedure. (b) Representative optical images of the stents and bile ducts after two months, with cross-sectional views. (c, d) Optical photographs of bile extract from rabbit bile duct after 2 months of experiments and measurements of its viscosity. (n=4). (e, f) Representative optical images of bile CAs and its corresponding quantitative analysis of CAs and SAs (n=4). *(*P < 0.05,* ***P < 0.01, ***P < 0.001, and ****P < 0.0001). ns, not significant.*


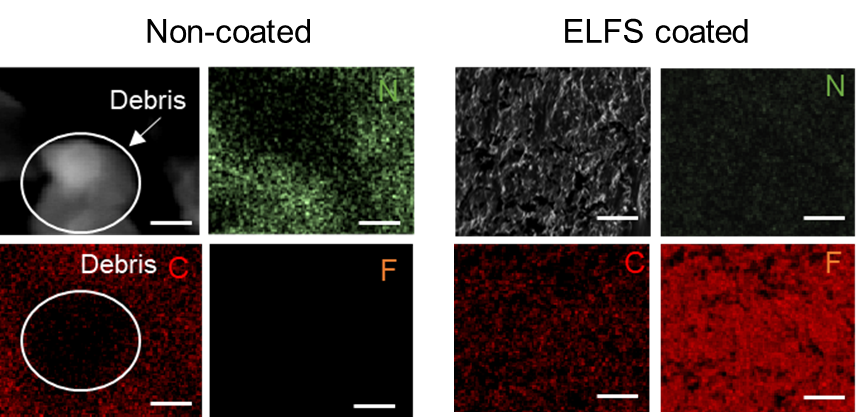


**Fig. S6.** Representative SEM images and corresponding EDS elemental maps (N, C, and F) of explanted stents after 2 months of implantation in the rabbit bile duct (scale bars, 20 μm).
